# p38- and MK2-dependent signalling promotes stress-induced centriolar satellite remodelling via 14-3-3-dependent sequestration of CEP131/AZI1

**DOI:** 10.1038/ncomms10075

**Published:** 2015-11-30

**Authors:** Maxim A. X. Tollenaere, Bine H. Villumsen, Melanie Blasius, Julie C. Nielsen, Sebastian A. Wagner, Jiri Bartek, Petra Beli, Niels Mailand, Simon Bekker-Jensen

**Affiliations:** 1 Ubiquitin Signaling Group, Protein Signaling Program, The Novo Nordisk Foundation Center for Protein Research, Faculty of Health and Medical Sciences, University of Copenhagen, Blegdamsvej 3B, Copenhagen DK-2200, Denmark; 2 Danish Cancer Society Research Center, Strandboulevarden 49, Copenhagen DK-2100, Denmark; 3 Department of Medicine, Hematology/Oncology, Goethe University Medical School, Theodor-Stern-Kai 7, Frankfurt DE-60590, Germany; 4 Division of Translational Medicine and Chemical Biology, Department of Medical Biochemistry and Biophysics, Karolinska Institute, Stockholm SE-17176, Sweden; 5 Institute of Molecular Biology, Ackermannweg 4, Mainz DE-55128, Germany

## Abstract

Centriolar satellites (CS) are small granular structures that cluster in the vicinity of centrosomes. CS are highly susceptible to stress stimuli, triggering abrupt displacement of key CS factors. Here we discover a linear p38-MK2-14-3-3 signalling pathway that specifically targets CEP131 to trigger CS remodelling after cell stress. We identify CEP131 as a substrate of the p38 effector kinase MK2 and pinpoint S47 and S78 as critical MK2 phosphorylation sites in CEP131. Ultraviolet-induced phosphorylation of these residues generates direct binding sites for 14-3-3 proteins, which sequester CEP131 in the cytoplasm to block formation of new CS, thereby leading to rapid depletion of these structures. Mutating S47 and S78 in CEP131 is sufficient to abolish stress-induced CS reorganization, demonstrating that CEP131 is the key regulatory target of MK2 and 14-3-3 in these structures. Our findings reveal the molecular mechanism underlying dynamic CS remodelling to modulate centrosome functions on cell stress.

## Abstract (web summary)

Centriolar satellites (CS) dynamically remodel in response to cellular stress. Here the authors describe a mechanism for stress-mediated remodelling, whereby CEP131 is phosphorylated downstream of p38, creating binding sites for 14-3-3 that lead to the sequestration of CEP131 in the cytoplasm and disassembly of CS.

## Graphical abstract

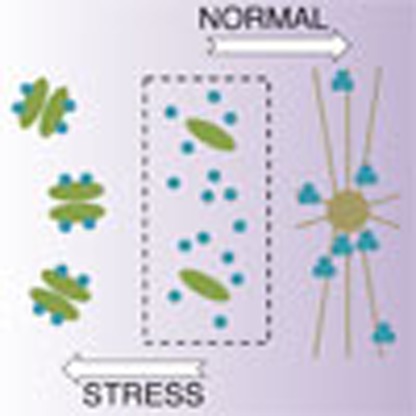

Centriolar satellites (CS) are small granular structures with a diameter of 70–100 nm that cluster in the vicinity of the centrosome^1^,^2^. CS are highly dynamic structures that travel along the microtubule network, and which can be readily observed by both electron and conventional light microscopy in the cytoplasm of mammalian interphase cells. By now, ∼30 proteins have been classified as bona fide CS components, many of which also localize to and function at the centrosome^2^. A growing list of CS factors is directly implicated in human pathologies, highlighting the clinical relevance of these structures^3^,^4^. In one prevalent model, CS function to maintain centrosomal proteostasis through the replenishment of factors via active microtubule-mediated transport to the centrosome or by acting as proximal and temporary storage containers^3^,^5^. Recent studies, however, have pointed to more elaborate functions of CS in promoting non-canonical roles of the centrosome such as primary cilium formation in quiescent cells and neurite outgrowth in neurons^3^,^4^,^6^. Despite such recent progress, our understanding of the functions, molecular composition and regulation of CS remains rudimentary.

We recently discovered a novel p38 Mitogen-Activated Protein Kinase (MAPK)-dependent signalling pathway that mediates abrupt collapse of CS on stress stimuli such as ultraviolet irradiation^7^. Accordingly, a range of CS components, including CEP131 (also known as AZI1), PCM1 and CEP290, undergo displacement from CS and disperse throughout the cytoplasm following exposure of cells to a number of different cellular stresses. Intriguingly, some CS components such as OFD1 do not change their localization under these conditions, suggesting that this novel stress response functions to rewire the composition and function of CS. Apart from the clear requirement of p38-dependent signalling, however, the molecular mechanisms underlying stress-induced CS reorganization are not known.

p38, similar to other MAPKs, acts to coordinate responses to environmental changes by regulating gene expression, cell growth, stress responses and apoptosis^8^,^9^. The MAPK p38 (of which the α isoform is the prevalent one in proliferating cells) is a central transducer of cellular stress pathways and is activated by a number of insults such as ultraviolet light, oxidative stress and heat or osmotic shock. In addition, p38 is activated by a number of extracellular signalling molecules such as growth factors, hormones and cytokines^9^. MAPKs rely on further signal propagation through direct activation of numerous downstream kinases, including members of the MAPK-activated protein kinase (MAPKAP) family, MSK and MNK kinases^10^. The combined activation of specific MAPKs and downstream effector kinases trigger elaborate signal transduction cascades targeting diverse cellular processes, allowing cells to respond appropriately to a wide range of cellular stresses. Interestingly, studies of the ultraviolet-induced DNA damage response (DDR) have uncovered a signalling network involving p38 and its downstream kinase MK2 (MAPKAPK2) that acts in parallel with classical DDR signalling pathways to regulate cell cycle progression, protein translation and RNA metabolism^11^,^12^,^13^,^14^.

In this study, we elucidated the molecular mechanism underlying ultraviolet-induced CS remodelling. We identify CEP131 as a major CS-associated substrate of p38-dependent, MK2-mediated phosphorylation on two defined residues and show that these modifications promote binding to 14-3-3 proteins, in turn leading to cytoplasmic sequestration of CEP131 and associated CS factors. Our findings reveal a critical role of the p38-MK2-14-3-3 signalling axis in promoting dynamic restructuring of CS in response to cellular stress.

## Results

### MK2 is required for CS remodelling

**Figure 1:**
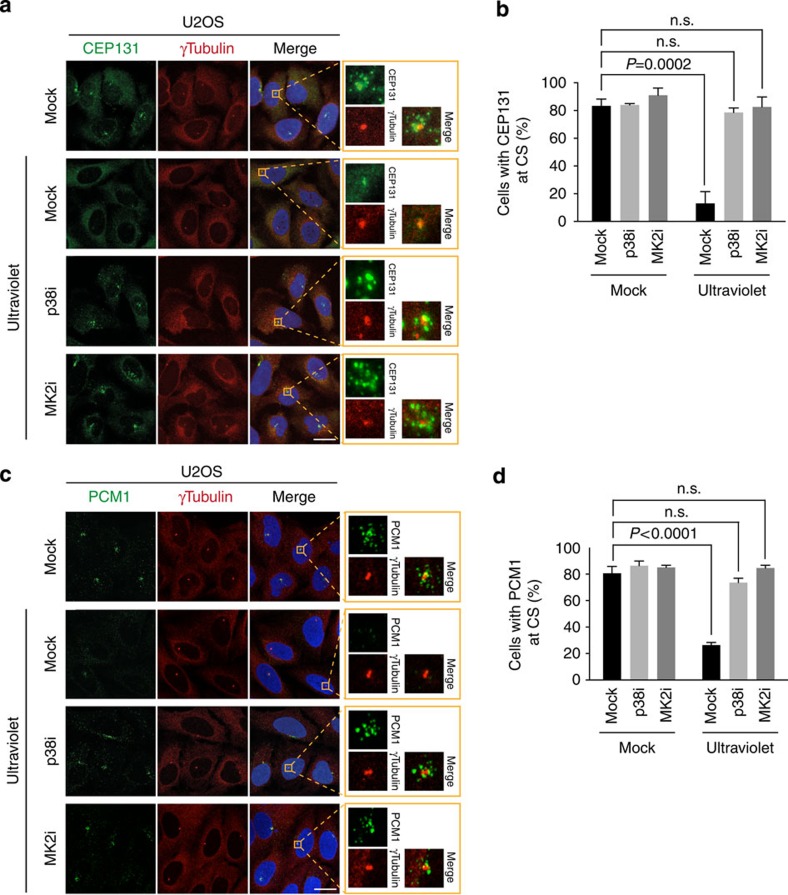
MK2 is required for stress-induced centriolar satellite reorganization. (**a**) U2OS cells were incubated with inhibitors against p38 (p38i) or MK2 (MK2i) for 1 h and exposed to ultraviolet irradiation as indicated. Cells were fixed 1 h later and co-immunostained with CEP131 and γ-tubulin antibodies. Inserts show magnified regions around the centrosomes. (**b**) Quantification of CEP131 localization to CS in cells treated as in **a**. At least 100 cells were scored per condition. Results (mean±s.d.) from three independent experiments are shown. *P* values were calculated from a one-tailed Student's *t*-test. (**c**) As in **a**, except that cells were co-immunostained with PCM1 and γ-tubulin antibodies. (**d**) Quantification of cells with CS localization of PCM1 analysed as in **b**. Scale bars, 10 μm.

MK2 is a major effector kinase downstream of p38, and previous work established an involvement of the p38-MK2 signalling axis in the regulation of processes such as cell cycle progression and RNA metabolism^11^,^14^,^15^. We have recently shown that ultraviolet-induced CS reorganization is fully dependent on p38 activity^7^, and we therefore reasoned that this process could also involve the MK2 kinase. To test this, we examined the localization of CS factors in response to ultraviolet irradiation in the presence or absence of small molecule inhibitors against p38 or MK2. In agreement with our previous findings, ultraviolet irradiation of human U2OS osteosarcoma cells led to a quantitative loss of CEP131, PCM1 and SSX2IP from CS in a p38-dependent manner (Fig. 1a–d and Supplementary Fig. 1a,b). The CS response was suppressed to a similar extent when MK2 function was abrogated by a specific chemical inhibitor, highlighting this kinase as an essential mediator of stress-induced CS remodelling downstream of p38. Importantly, this was not due to destabilization of any of a number of CS factors tested (Supplementary Fig. 1c). We made similar observations in immortalized human retinal pigment epithelial (RPE1) diploid cells (Supplementary Fig. 2), suggesting that stress-induced CS remodelling via p38 and MK2 is a general feature of human cells.

### MK2 phosphorylates CEP131 in response to ultraviolet

**Figure 2:**
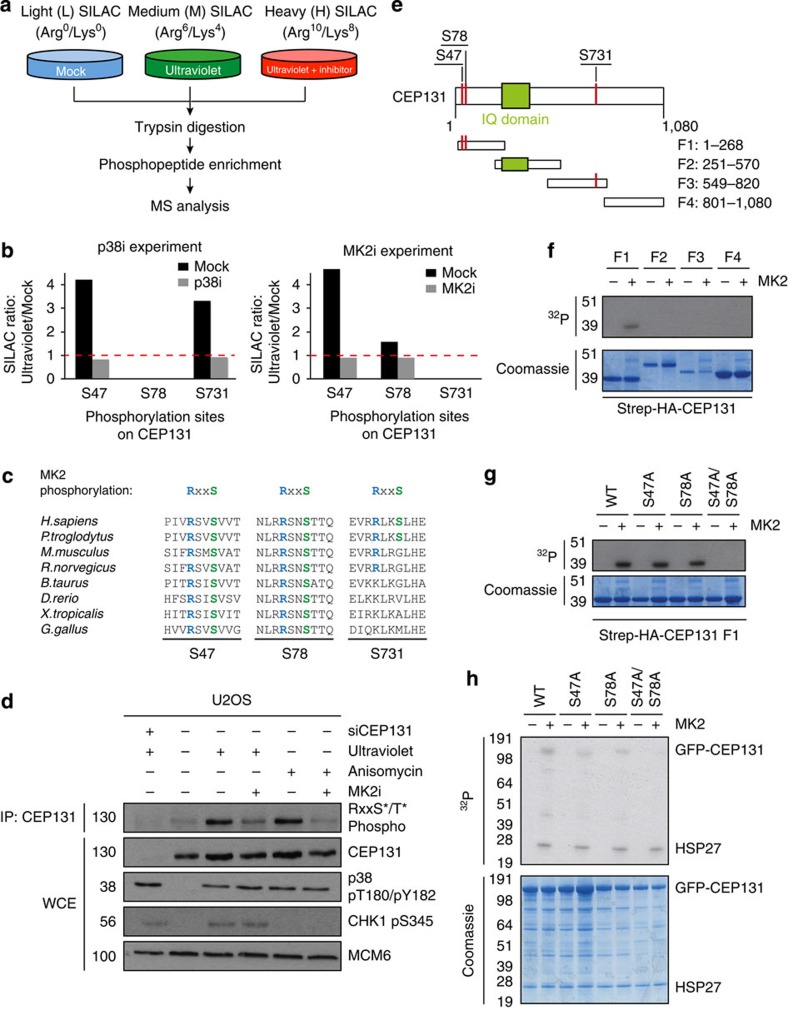
MK2 phosphorylates CEP131 on S47 and S78. (**a**) Outline of SILAC-based phosphoproteomics experiment. U2OS cells were isotope-labelled in culture with light, medium and heavy amino acids and exposed to kinase inhibitors and ultraviolet irradiation for 1 h as indicated. Cell lysates were pooled, processed and analysed by MS. (**b**) Ultraviolet-regulated phosphorylation sites in CEP131 responsive to inhibition of p38 (p38i; left) or MK2 (MK2i; right), identified and quantified by SILAC and phosphoproteomic analysis. (**c**) Alignment of CEP131 amino-acid sequences surrounding three putative MK2 phosphorylation sites (S47, S78 and S731, highlighted in green) from different organisms. (**d**) U2OS cells were incubated for 1 h with MK2 inhibitor (MK2i) and exposed to ultraviolet irradiation or Anisomycin for 1 h as indicated. Input lysates (WCE) and immunoprecipitated (IP) CEP131 were analysed by immunoblotting with the indicated antibodies. (**e**) Schematic representation of human CEP131 with annotated domains, identified p38- and MK2-responsive phosphorylation sites and overlapping Strep-HA-CEP131 fragments used for *in vitro* kinase assays. (**f**) Overlapping Strep-HA-tagged CEP131 fragments (F1–F4) were expressed in U2OS cells, purified on streptavidin beads and subjected to phosphorylation by MK2 *in vitro* in the presence of radioactive ATP (^32^P). Incorporation of radioactive phosphate was assessed using autoradiography. (**g**) As in **f**, using indicated point mutants of Strep-HA-CEP131-F1 constructs. (**h**) WT and mutant versions of full-length GFP-CEP131 were purified from cells using GFP-Trap beads and subjected to MK2 phosphorylation as in **f**. Recombinant HSP27 was added to the reactions as a positive control for MK2 phosphorylation. Unprocessed original scans of western blots are shown in Supplementary Fig. 11.

Prompted by these findings, we asked whether MK2 phosphorylates any of the known CS factors. To this end, we used Stable Isotope Labelling with Amino acids in Cell culture and mass spectrometry (SILAC/MS) to map p38- and/or MK2-dependent phosphorylation events in response to ultraviolet (Fig. 2a). Using this approach, where a SILAC ratio >1 indicates an enrichment of the phosphorylated state, we identified three serine residues (S47, S78 and S731) in CEP131, whose phosphorylation increased on ultraviolet irradiation in a p38- and/or MK2-dependent manner (Fig. 2b). Notably, the amino-acid sequences surrounding each of the MK2-regulated serine residues corresponded to the known minimal consensus for MK2 phosphorylation (RXXpS/T)^11^, suggesting that S47, S78 and S731 are direct targets of MK2-dependent phosphorylation (Fig. 2c). Consistently, endogenous CEP131 was immunoreactive with a generic phospho-specific antibody recognizing phosphorylated RXXpS/T peptides, and this phospho-signal increased after exposure to ultraviolet as well as the p38-activating compound anisomycin in a manner dependent on MK2 activity (Fig. 2d). In the case of both S47 and S78, but not S731, these motifs displayed a high degree of evolutionary conservation (Fig. 2c), indicating that S47 and S78 are functionally important substrates of MK2 phosphorylation. Importantly, we did not find phosphorylation sites in any other known CS components that robustly matched all of these criteria (Supplementary Data 1), suggesting that CEP131 might be a key target of p38/MK2-dependent phosphorylation in the stress-induced response leading to CS reorganization.

To test whether MK2 phosphorylates CEP131 directly, we generated and purified four overlapping CEP131 fragments (Fig. 2e), which were then subjected to MK2 phosphorylation *in vitro*. Strikingly, only the N-terminal fragment (CEP131-F1) containing S47 and S78, but not the region containing S731 (CEP131-F3), underwent phosphorylation by MK2 under these conditions (Fig. 2e,f). We then made alanine substitutions of S47 and/or S78 in F1 and examined the impact of these mutations on MK2 phosphorylation *in vitro*. We found that, while individual mutation of S47 or S78 partially reduced the phosphorylation of CEP131-F1 by MK2, the double S47A/S78A mutant was completely refractory to MK2-dependent phosphorylation (Fig. 2g), demonstrating that both of these residues are targeted by MK2, in agreement with our MS data. Similarly, combined mutation of S47 and S78 was sufficient to abolish phosphorylation of full-length CEP131 by MK2 (Fig. 2h), suggesting that S47 and S78 represent the major sites of MK2 phosphorylation in CEP131. These findings establish a direct role for MK2 in the stress-induced response leading to CS reorganization and suggest that CEP131 is a major CS-associated substrate of this kinase.

### MK2 phosphorylation of CEP131 promotes 14-3-3 binding

**Figure 3:**
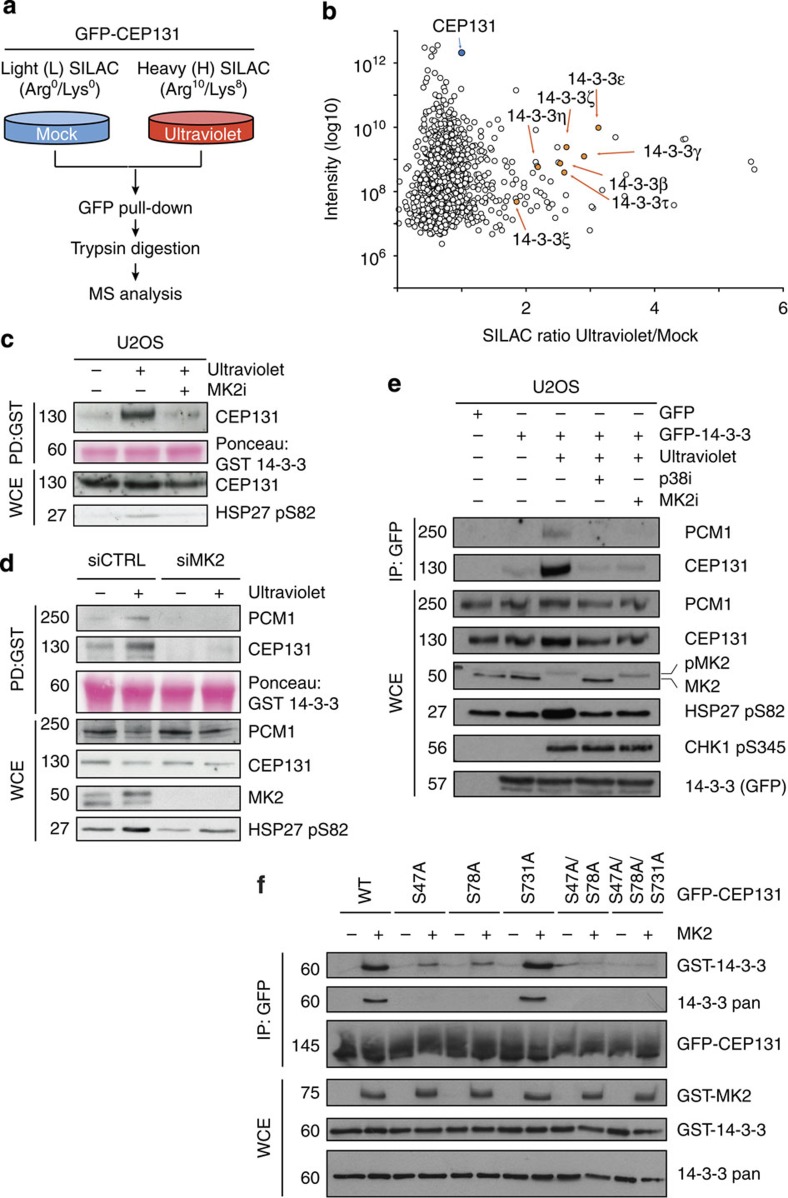
MK2-mediated phosphorylation of CEP131 on S47 and S78 promotes 14-3-3 binding. (**a**) Outline of SILAC-based and proteomic analysis of ultraviolet-regulated CEP131 interactome. U2OS/GFP-CEP131 cells were isotope-labelled in culture with light and heavy amino acids and exposed to ultraviolet irradiation as indicated and collected 1 h later. GFP-CEP131 and associated proteins were purified on GFP-Trap agarose and analysed using MS. (**b**) SILAC ratios from **a** represented as a scatter plot. The bait (CEP131) is highlighted in blue and ultraviolet-enriched 14-3-3 isoforms in orange. Data were reanalysed from a previous study^7^. (**c**) Lysates from U2OS cells treated with MK2 inhibitor and ultraviolet as indicated were incubated with recombinant GST-14-3-3. Input lysates and GST-bound material were analysed by immunoblotting with the indicated antibodies. (**d**) U2OS transfected with non-targeting control (CTRL) or MK2 siRNAs were processed as in **c**. (**e**) Extracts of U2OS cells transfected with GFP-14-3-3 construct and incubated with p38 or MK2 inhibitors for 1 h before ultraviolet irradiation as indicated were subjected to GFP IP. IPs and input lysates were analysed by immunoblotting with the indicated antibodies. (**f**) WT and mutant versions of GFP-CEP131 were expressed in U2OS cells, isolated on GFP-Trap agarose and treated with lambda phosphatase before *in vitro* phosphorylation by recombinant GST-MK2. After washing, beads were incubated with GST-14-3-3, and CEP131/14-3-3 binding was analysed by immunoblotting with the indicated antibodies. Unprocessed original scans of western blots are shown in Supplementary Fig. 11.

In our previous work, we used SILAC/MS to pinpoint cellular-binding partners of CEP131 (ref. 7). Interestingly, on reanalysis of these data, we noticed that all members of the 14-3-3 family of proteins displayed enhanced interaction with CEP131 in response to ultraviolet irradiation, as indicated by a SILAC ratio >1 when comparing heavy (H) versus light (L) peptides (Fig. 3a,b). 14-3-3 proteins form homo- or heterodimeric complexes that engage in phospho-dependent interactions with a range of client proteins, in many cases inhibiting specific protein–protein or protein–RNA interactions, modulating enzymatic activity or sequestering binding partners away from their normal sites of action^13^,^16^,^17^,^18^. Given the striking overlap between the MK2 phosphorylation site consensus (RXXpS/T) and reported 14-3-3-binding motifs (RSXpSXP and RXY/FXpSXP)^19^,^20^,^21^, we surmised that phosphorylation of CEP131 by MK2 in response to ultraviolet could promote binding to 14-3-3 proteins. To test this, we analysed binding of recombinant glutathione *S*-transferase (GST)-tagged 14-3-3 to CEP131 in cell extracts. Consistent with our MS data (Fig. 3b), we found that endogenous CEP131 bound to GST-14-3-3 in a manner that was strongly enhanced by ultraviolet (Fig. 3c). Importantly, the CEP131/14-3-3 interaction was abolished when MK2 was inhibited or depleted (Fig. 3c,d), suggesting that MK2 kinase activity plays a pivotal role in promoting interaction between CEP131 and 14-3-3 proteins. We verified the interaction between 14-3-3 and CEP131 in cells, where GFP-tagged 14-3-3 interacted with endogenous CEP131 in a ultraviolet-induced manner that was strictly dependent on both p38 and MK2 activity (Fig. 3e). Intriguingly, another CS factor, PCM1, also co-purified with GFP-14-3-3 and CEP131 in these experiments (Fig. 3d,e), raising the possibility that CEP131 could bridge interactions between 14-3-3 and other CS components.

To further test the hypothesis that MK2-dependent phosphorylation of CEP131 promotes its association with 14-3-3, we sought to reconstitute MK2-mediated binding between these factors *in vitro*. To this end, we immunopurified wild-type (WT) or mutant forms of GFP-CEP131 expressed in cells, subjected these proteins to phosphorylation by MK2 and assessed their interactions with recombinant GST-14-3-3. Strikingly, under these conditions, interaction between 14-3-3 and CEP131 only occurred when CEP131 had been phosphorylated by MK2 (Fig. 3f), further confirming that MK2-dependent phosphorylation of CEP131 is an important trigger of its binding to 14-3-3. Using this experimental set-up, we analysed whether the integrity of the MK2 phosphorylation sites we mapped in CEP131 (S47 and S78) was required for binding to 14-3-3. Indeed, single S47A or S78A mutations led to a robustly decreased interaction between CEP131 and 14-3-3, while the association was completely abolished on simultaneous mutation of both MK2 phosphorylation sites (Fig. 3f). In contrast, mutation of S731 had no effect on the interaction between CEP131 and 14-3-3 (Fig. 3f). We conclude from these data that MK2-dependent phosphorylation of CEP131 on S47 and S78 generates binding sites for 14-3-3 proteins in response to ultraviolet.

### 14-3-3 promotes cytoplasmic localization of CS factors

**Figure 4:**
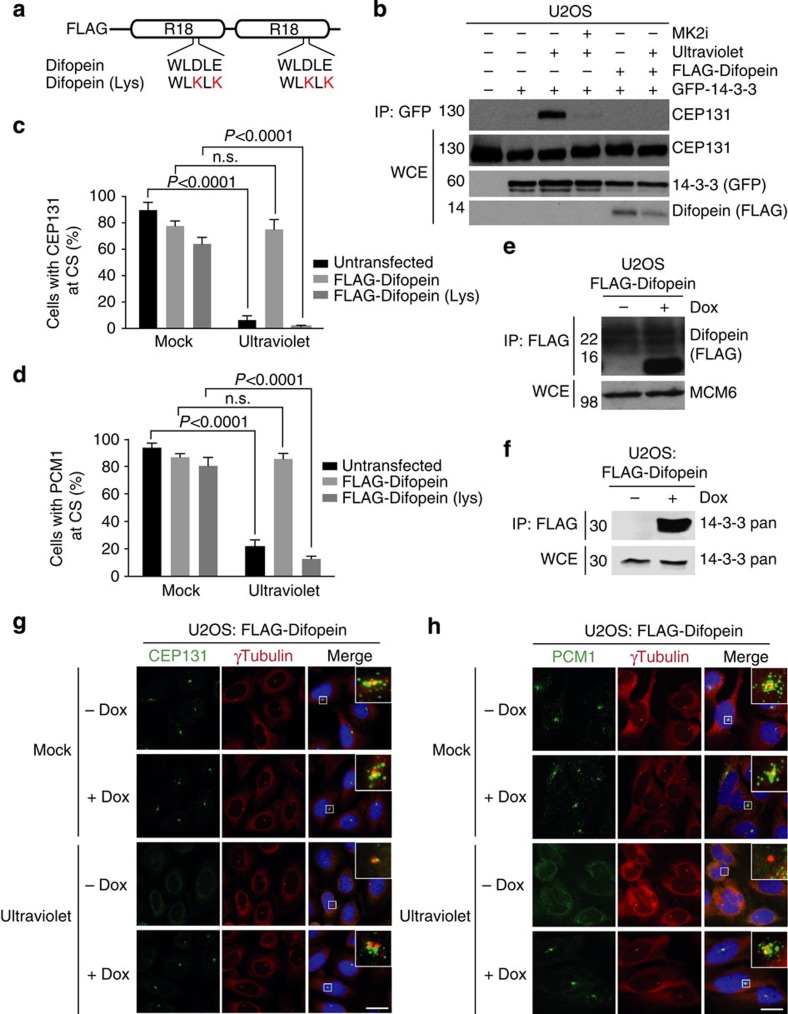
14-3-3 proteins are required for stress-induced displacement of centriolar satellite factors. (**a**) Schematic representation of unmodified and 14-3-3-binding-deficient (Lys) FLAG-Difopein constructs. (**b**) U2OS cells co-transfected with GFP-14-3-3 and FLAG-Difopein and exposed to ultraviolet irradiation, as indicated, were lysed and subjected to GFP pull-downs. Binding between GFP-14-3-3 and endogenous CEP131 was analysed by immunoblotting with the indicated antibodies. (**c**) Quantification of cells with CS localization of CEP131 after ultraviolet irradiation in cells transfected with FLAG-Difopein or FLAG-Difopein (Lys) constructs. At least 100 cells with low to moderate FLAG staining were scored per condition. Results (mean±s.d.) from three independent experiments are shown. *P* values were calculated from a one-tailed Student's *t*-test. Representative images are shown in Supplementary Fig. 3a. (**d**) As in **c**, except that CS were counterstained for PCM1. Representative images are shown in Supplementary Fig. 3b. (**e**) U2OS/FLAG-Difopein cells incubated or not with Doxycycline (Dox) for 24 h were lysed and subjected to FLAG immunoprecipitation followed by immunoblotting with the indicated antibodies. (**f**) As in **e**, except that an antibody broadly recognizing 14-3-3 isoforms (14-3-3 pan) was used for immunoblotting. (**g**) U2OS/FLAG-Difopein cells grown in the presence or absence of Doxycycline and exposed to ultraviolet irradiation as indicated, were fixed and co-immunostained with antibodies to CEP131 and γ-tubulin. Inserts show magnified regions around the centrosomes. (**h**) As in **g**, except that cells were co-immunostained with PCM1 and γ-tubulin antibodies. Scale bars, 10 μm. Unprocessed original scans of western blots are shown in Supplementary Fig. 11.

The findings above suggested that MK2-dependent binding of 14-3-3 proteins to CEP131 might play an active role in promoting ultraviolet-induced CS restructuring. To test this, we took advantage of the peptide-based 14-3-3 inhibitor Difopein (Dimeric Fourteen-Three-Three-Peptide Inhibitor), which binds with high affinity to all 14-3-3 isoforms in a phospho-independent manner, suppressing their functionality (Fig. 4a)^22^. Expression of FLAG-Difopein quantitatively suppressed the ability of GFP-14-3-3 to interact with endogenous CEP131 in ultraviolet-irradiated U2OS cells (Fig. 4b), confirming that this construct efficiently blocks 14-3-3/protein interactions in cells. To determine whether 14-3-3 proteins are required for stress-induced CS remodelling, we assessed the subcellular localization of CEP131 and PCM1 in cells overexpressing FLAG-Difopein or a modified version (Difopein (Lys)) incapable of binding to 14-3-3 because of mutation of critical acidic residues in the two interaction domains (Fig. 4a)^22^,^23^. We found that moderate expression of Difopein, but not the Lys mutant, completely blocked the ultraviolet-induced displacement of CEP131 and PCM1 from CS (Fig. 4c,d and Supplementary Fig. 3a–c), while the overall appearance of CS in unstressed cells was not overtly affected. We further confirmed these observations in stable cell lines expressing FLAG-Difopein at low and uniform levels in an inducible manner (Fig. 4e and Supplementary Fig. 4a). Under these conditions, FLAG-Difopein bound strongly to endogenous 14-3-3 (Fig. 4f) and completely blocked ultraviolet-induced dispersal of the CS components CEP131, PCM1 and SSX2IP (Fig. 4g,h and Supplementary Fig. 4b). Importantly, induction of FLAG-Difopein did not interfere with ultraviolet-induced activation of p38 or MK2 (Supplementary Fig. 4c), thus indicating that 14-3-3 proteins function downstream of these kinases in promoting CS reorganization. We conclude that a linear p38-MK2-14-3-3 signalling cascade is responsible for disassembling CS in stressed cells by sequestering CEP131 and associated components in the cytoplasm.

### 14-3-3-CEP131 binding underlies stress-induced CS remodelling

**Figure 5:**
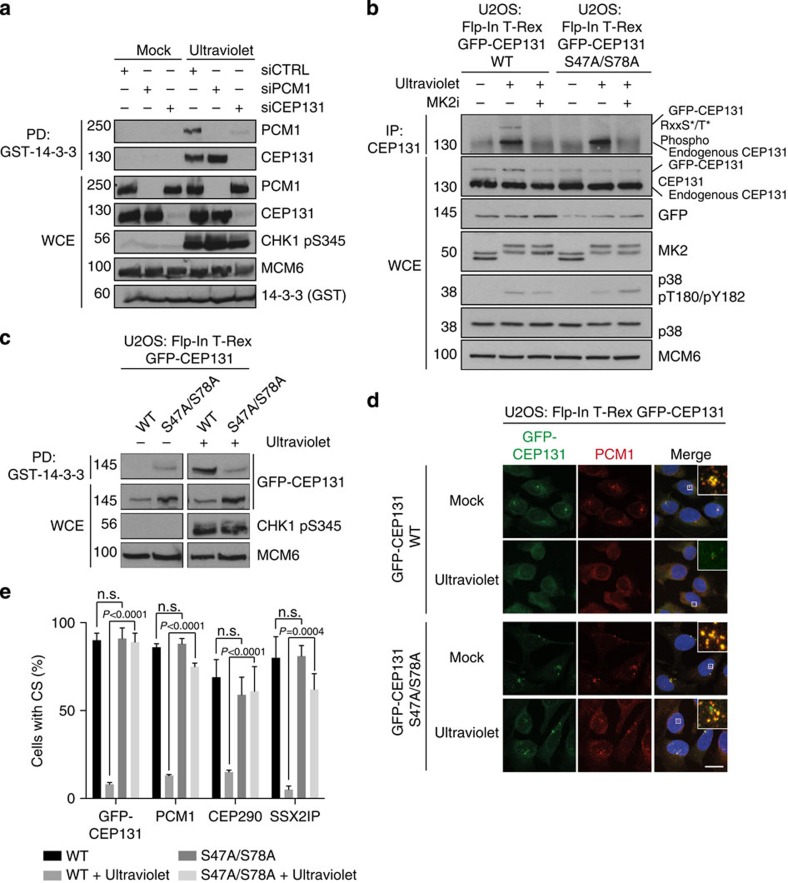
Phosphorylation of CEP131 on S47 and S78 is required for 14-3-3-dependent centriolar satellite remodelling upon cell stress. (**a**) Extracts of U2OS cells transfected with indicated siRNAs and exposed to ultraviolet irradiation or not were lysed and incubated with recombinant GST-14-3-3. Input lysates and GST pull-downs were analysed by immunoblotting with the indicated antibodies. (**b**) U2OS: Flp-In T-Rex GFP-CEP131 WT and mutant (S47A/S78A) cells were pre-treated with MK2 inhibitor and exposed to ultraviolet irradiation (1 h), as indicated. Input lysates and CEP131 IP were analysed by immunoblotting with the indicated antibodies. (**c**) Cells from **b** were exposed to ultraviolet irradiation as indicated. Cells were lysed 1 h after ultraviolet irradiation and lysates were incubated with GST-14-3-3. Input lysates and GST-co-purified material were analysed by immunoblotting with the indicated antibodies. The split panels originate from the same membrane with identical exposure time. (**d**) Cells from **b** were fixed and immunostained with antibodies against PCM1. Scale bar, 10 μm. Inserts show magnified regions around the centrosomes. (**e**) Quantification of cells with CS localization of GFP-CEP131, PCM1, SSX2IP and CEP290 with or without ultraviolet irradiation. At least 100 cells were scored per condition and results reflect the mean (±s.d.) of three independent experiments. *P* values were calculated from a one-tailed Student's *t*-test. Representative images of SSX2IP and CEP290 are shown in Supplementary Fig. 5a,b. Unprocessed original scans of western blots are shown in Supplementary Fig. 11.

While both CEP131 and PCM1 interact with 14-3-3 in an MK2-dependent manner (Fig. 3d,e), the apparent absence of MK2 phosphorylation sites in CS components other than CEP131 (Supplementary Data 1) suggested that this protein could provide a direct handle for 14-3-3-mediated reconfiguration of these structures after cell stress. To address this, we asked whether CEP131 was required for binding of PCM1 to 14-3-3 and *vice versa*. We found that, while CEP131/14-3-3 binding occurred independently of PCM1, the interaction between PCM1 and 14-3-3 was strongly impaired in CEP131-depleted cells (Fig. 5a). This supports the notion that CEP131 is a major CS-associated factor capable of interacting with 14-3-3, and that interactions between 14-3-3 to other CS components such as PCM1 are likely indirect and bridged by CEP131. To further test this model, we generated cell lines expressing low levels of WT or MK2 phosphorylation-deficient (S47A/S78A) forms of GFP-CEP131. Consistent with our previous observations, only GFP-CEP131 WT, but not the S47A/S78A mutant, reacted with an antibody specific to phosphorylated RXXpS/T peptides and co-precipitated with GST-14-3-3 in a ultraviolet-inducible manner in these cell lines (Fig. 5b,c).

To test whether mutation of the MK2 phosphorylation sites in GFP-CEP131 interferes with stress-induced CS remodelling, we next examined the subcellular localization of CS components in these cell lines (Fig. 5d,e). Whereas cells ectopically expressing GFP-CEP131 WT showed a normal stress-induced CS response characterized by ultraviolet-induced displacement of GFP-CEP131 and endogenous PCM1, expression of the MK2 phosphorylation- and 14-3-3-binding-deficient S47A/S78A allele completely abrogated this response (Fig. 5d,e). The ultraviolet-induced displacement of other CS factors such as SSX2IP and CEP290 was also fully suppressed in cells expressing S47A/S78A, but not WT, GFP-CEP131 (Fig. 5e and Supplementary Fig. 5a,b). Importantly, these effects could not be attributed to a defect in p38 pathway activation as measured by ultraviolet-induced phosphorylation of p38, MK2 and HSP27 (Fig. 5b and Supplementary Fig. 5c). The persistent CS localization of all of these factors in ultraviolet-irradiated cells expressing CEP131 S47A/S78A suggests that CEP131 phosphorylation by MK2 and ensuing 14-3-3 binding is essential for the displacement of CS-associated factors on cell stress. We also established a cell line expressing an allele of GFP-CEP131 with acidic residues at positions 47 and 78 (S47E/S78E); however, this mutant behaved essentially like the alanine mutant, suggesting that phosphate groups are required to mediate the interaction with 14-3-3 (Supplementary Fig. 6).

### CEP131 (S47A/S78A) supports CS dynamics in unstressed cells

Ectopic expression of CS components such as CEP131 is challenging because of their inherent propensity to assemble into large and insoluble protein aggregates (our unpublished observations). To address these concerns, and to further characterize the impact of p38-MK2 signalling on CS biology, we carefully compared the behaviour of WT and mutant GFP-CEP131 with that of the endogenous protein. Importantly, in our cell lines both WT and mutant GFP-CEP131 required PCM1 for targeting to CS (but still displayed PCM1-independent affinity for the centrosome^24^,^25^; Supplementary Fig. 7a), and this localization was lost on mitosis^26^,^27^ (Supplementary Fig. 7b). In addition, time-lapse studies showed that, consistent with the reported dynamic behaviour of CS in live cells^28^, all of the previously reported behaviours of CS, including fusion and dissociation events and step-wise transport towards the centrosome along microtubules could be observed in cells expressing WT and mutant GFP-CEP131 (Supplementary Movies 1 and 2). This demonstrates that the GFP-CEP131-positive granules in our cell lines recapitulate all the hallmarks of physiological CS.

Prompted by these observations, we utilized the cell lines stably expressing GFP-CEP131 constructs to replace endogenous CEP131 with near-physiological levels of the GFP-CEP131 transgenes, by combining short interfering RNA (siRNA)-mediated knockdown and carefully optimized dosing of low concentrations of doxycycline (Supplementary Fig. 8a). Consistent with our previous observations, only GFP-CEP131 WT, but not the S47A/S78A mutant, efficiently co-purified with GST-14-3-3 in a ultraviolet-dependent manner (Supplementary Fig. 8b) and supported the dispersal of GFP-CEP131 and PCM1 from CS (Supplementary Fig. 8c). Thus, the CEP131 S47A/S78A mutant is fully proficient in supporting all known features of CS biology except their stress-induced compositional reorganization. We therefore conclude that MK2-dependent phosphorylation of CEP131 at S47 and S78 and the ensuing binding of 14-3-3 proteins play an essential role in triggering stress-induced remodelling of CS.

### 14-3-3–CEP131 interaction blocks formation of CS

**Figure 6:**
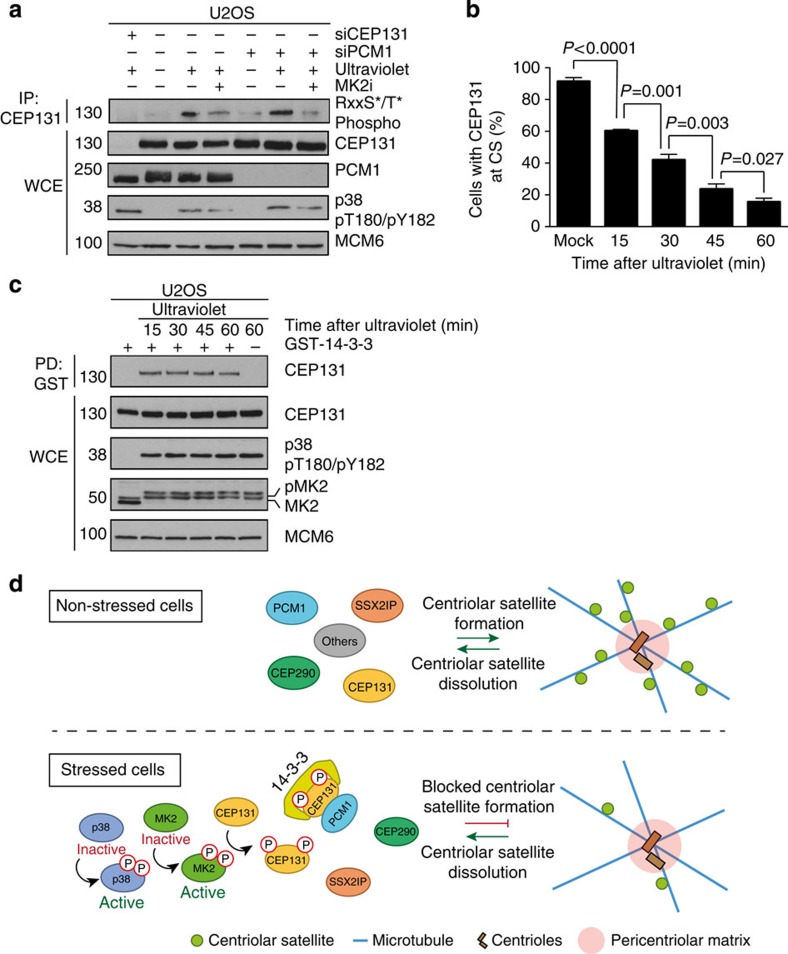
MK2- and 14-3-3-dependent CEP131 sequestration blocks CS formation after cell stress. (**a**) U2OS cells were transfected with indicated siRNAs and exposed to MK2 inhibitor for 1 h and ultraviolet irradiation (1 h) as indicated. Input lysates and CEP131 IP were analysed by immunoblotting with the indicated antibodies. (**b**) U2OS cells were exposed to ultraviolet irradiation, fixed at the indicated time points and co-immunostained with CEP131 and γ-tubulin antibodies. At least 100 cells were scored per condition. Results (mean±s.d.) from three independent experiments are shown. *P* values were calculated from a one-tailed Student's *t*-test. Representative images are shown in Supplementary Fig. 10. (**c**) Lysates from cells in **b** were incubated with GST-14-3-3. Input lysates and GST-co-purified material were analysed by immunoblotting with the indicated antibodies. (**d**) Model of stress-induced CS remodelling. In unstressed cells (upper panel), CS are maintained through an equilibrium between formation and dissolution events. On ultraviolet irradiation and other stress stimuli (lower panel), p38 kinase is activated, resulting in phosphorylation and activation of its downstream kinase MK2. MK2 directly phosphorylates two residues on CEP131, S47 and S78, to create dual binding sites for 14-3-3 proteins. The resulting interaction mediates cytoplasmic sequestration of CEP131 that blocks *de novo* CS formation, leading to a net progressive loss of CS in cells. Unprocessed original scans of western blots are shown in Supplementary Fig. 11.

CS are dynamic structures, which continuously form and dissociate as they travel towards the centrosome along the microtubule network^28^ (Supplementary Movies 1 and 2). Thus, CEP131 and other CS components continuously shuttle between mobile cytoplasmic and immobile CS-bound pools. To better understand why 14-3-3 binding to CEP131 results in CS depletion, we considered two possible scenarios underlying this phenomenon: (1) MK2 phosphorylation and 14-3-3 binding target the CS-resident CEP131 pool and extract the protein from this locale or (2) the same events target CEP131 in the cytoplasm to block *de novo* formation of CS after stress. To test the first hypothesis, we carefully assayed for localization of MK2 or 14-3-3 to CS during the ultraviolet-induced stress response. However, we failed to observe detectable accumulation of these proteins at CS (Supplementary Fig. 9a,b). This was also the case for MK2 when activated by p38 after ultraviolet but chemically inhibited, so that it was unable to promote CEP131 phosphorylation and CS remodelling (Supplementary Fig. 9b). In addition, we found that MK2-dependent phosphorylation of CEP131 and ensuing 14-3-3 binding were independent of PCM1 and thus did not require localization of CEP131 to CS (Figs 5a and 6a). These observations argued against a model, in which CEP131 and other factors are directly extracted from CS on cell stress and prompted us to examine the kinetics of CS remodelling. Remarkably, we found that ultraviolet-induced CS remodelling, as measured by the presence of CEP131-positive granules in cells, progressively evolved to reach a peak around 1 h after ultraviolet treatment, suggesting that this response is caused by a gradual loss of intact CS rather than by abrupt stress-induced collapse of these structures (Fig. 6b and Supplementary Fig. 10). Importantly, the kinetics of CEP131 loss from CS was strikingly different from that of p38 and MK2 activation and 14-3-3/CEP131 complex formation, which was maximal already 15 min after ultraviolet (Fig. 6c). On the basis of these observations, we propose that phosphorylation and cytoplasmic sequestration of CEP131 occur rapidly after ultraviolet to block the formation of new CS, thereby leading to a net progressive depletion of these structures after stress.

## Discussion

In this study, we have elucidated the molecular mechanism governing p38-dependent CS reorganization during cellular stress responses (Fig. 6d). We have shown that this process is fully dependent on the activity of MK2, a key effector kinase downstream of p38, and that MK2-mediated phosphorylation of the CS factor CEP131 generates stress-induced binding sites for 14-3-3 proteins, leading to sequestration of CEP131 in the cytoplasm. Several 14-3-3 ligands have been shown to contain dual 14-3-3 interaction motifs, required to initiate and stabilize the interaction, respectively^14^,^17^,^19^,^29^. This is also likely to be the case for CEP131, which is phosphorylated by MK2 on two residues (S47 and S78), both of which are required for efficient association with 14-3-3. While 14-3-3 directly interacts with CEP131, the interaction with PCM1 appears to be bridged by CEP131 (Fig. 5a). In addition, the dispersal of both CEP131 as well as other CS components including PCM1 can be completely abrogated by mutating S47 and S78 in CEP131 (Fig. 5d,e and Supplementary Fig. 5a,b). Our data therefore suggest that phosphorylated CEP131 is likely to be the main, if not the only, CS-associated factor capable of interacting with 14-3-3 proteins, and that this interaction underlies the overall reorganization of CS following cell stress, characterized by the concomitant loss of other CS factors including PCM1, CEP290 and SSX2IP from these structures. Our experiments further suggest that binding of 14-3-3 to CEP131 does not disrupt existing CS, but rather prevents the *de novo* formation of new CS, thereby causing a net progressive loss of these structures after stress (Fig. 6a–c and Supplementary Figs 9 and 10). These conclusions are in line with other studies, where 14-3-3 binding was found to prevent the integration of client proteins into larger protein complexes^30^. This scenario also accounts well for the observation that stress-induced CS depletion can be completely blocked by the expression of subendogenous levels of a GFP-CEP131 construct that is refractory to MK2 phosphorylation (Fig. 5b,d and Supplementary Fig. 5a,b). This phosphorylation-deficient mutant still supports the formation of new CS even when the endogenous pool of CEP131 is sequestered after cell stress, thus maintaining the pool of CS under these conditions.

Most of the known CS factors, including CEP131 and SSX2IP, display a dual localization pattern, associating independently with both CS and the centrosome^31^,^32^. While a subset of these factors are lost from CS on ultraviolet and other stresses, the centrosomal localization of the same factors is not affected by such insults. This raises the important question of what mechanism(s) protects the centrosomal pool of these proteins from MK2 phosphorylation and 14-3-3-dependent sequestration. Given our observation that these events occur in the cytoplasm, attractive hypotheses are that centrosomal CEP131 is either refractory to MK2-dependent phosphorylation or that the ensuing 14-3-3 binding does not prevent CEP131 association with this structure. Intriguingly, similar observations were reported for the CS factor OFD1 (ref. 33). Despite the removal of OFD1 from CS by autophagy on ciliogenesis, the centrosomal fraction of OFD1 remains intact during these processes and is even crucial for primary cilium formation. These findings suggest that, while proteins residing in CS are exquisitely prone to regulation by a variety of cell stress conditions, perhaps as a result of the continuous and dynamic formation and dissolution of these structures, the same factors may be shielded from such intervention through unknown mechanisms when associated with centrosomes.

Prior work has uncovered other pathways that utilize p38-/MK2-dependent phosphorylation and subsequent 14-3-3 binding to modulate biological processes during stress responses such as that induced by ultraviolet light. A prime example of this is the contribution to cell cycle arrest mediated via p38-, MK2- and 14-3-3-dependent cytoplasmic sequestration of the mitosis-promoting phosphatases CDC25B and CDC25C (refs 11, 15, 34). Activation of p38 and MK2 prominently has an impact on the stability of a large number of RNAs to change the protein translation landscape of stressed cells. A general mechanism appears to be the targeting of proteins that bind AU-rich elements (AREs) and mediate either stabilization or degradation of the client mRNAs. For instance, MK2 phosphorylation affecting both negative and positive regulators of *GADD45α* mRNA stability collectively results in a ultraviolet-induced increase in GADD45α expression levels^11^,^35^,^36^. A similar pathway was reported to act on the ARE-binding factor TTP during an arsenite-induced stress response. Here MK2 phosphorylation and subsequent 14-3-3 binding excludes TTP from association with stress granules on arsenite treatment, protecting TTP-bound ARE-containing transcripts from degradation^37^. Finally, a p38-MK2-14-3-3-dependent pathway was recently linked to the regulation of RNA processing during the DDR and/or stress responses^14^. To this end, it was shown that RBM7, a component of the nuclear exosome targeting complex, is phosphorylated by MK2 to promote 14-3-3 binding, inhibiting the degradation of a subset of RNA molecules in the exosome^14^. Collectively, a common p38-MK2-14-3-3 pathway thus emerges as a central signalling module employed during cellular stress responses that have an impact on a variety of biological processes such as cell cycle progression, protein synthesis and RNA processing^10^,^38^. While such events channelled via p38 and MK2 after ultraviolet have mostly been ascribed to the DNA-damaging effects of ultraviolet, we consider it likely that they may rather reflect a general response to cell stress induced by the highly pleiotropic damaging effects on cellular macromolecules caused by ultraviolet irradiation.

The work presented here, together with our previous findings^7^, shows that intact CS are largely lost from cells during p38-mediated stress responses. Intriguingly, similar dynamic remodelling of CS was shown to underlie a variety of centrosome-associated biological responses. For example, primary cilium formation is associated with the exclusion of BBS4 from CS and degradation of CS-associated OFD1 (refs 4, 33). In addition, proliferation of CS, as visualized by PCM1- and centrin-positive granules, was shown to precede and be functionally linked to amplification of centrosomes at prolonged times after exposure of cells to DNA-damaging agents^39^,^40^. Together, these studies highlight CS as inherently dynamic structures, whose context-specific remodelling can have diverse impacts on a variety of cellular processes. Thus, CS are emerging as central regulatory hubs for a host of cellular responses that can alter the composition and/or stability of these structures, with poorly understood functional consequences.

In conclusion, our study adds stress-induced reorganization of CS to the growing cellular repertoire of p38-MK2-14-3-3 effector pathways. Despite these insights, we are still far from understanding the exact biological ramifications of this novel aspect of cellular stress responses, hampered to a large extent by the limited insights into the cellular functions of CS and their underlying mechanisms. Studies over the last decade have suggested roles for CS in ciliogenesis^4^,^33^,^41^, mitotic spindle pole maintenance^32^, centrosome maintenance/duplication^25^,^31^ and even neurogenesis^6^,^24^. In our previous work, we reported that ultraviolet irradiation induces cilium formation in RPE1 cells in a partially p38-dependent manner^7^. It is thus formally possible that the p38-/MK2-mediated pathway described in the present study is involved in this response. Future work will be required to further elucidate the supporting roles of CS for centrosome-associated functions and shed light on how such processes are modulated in response to cellular stress to protect cellular homeostasis.

## Methods

### Plasmids and siRNA

Full-length *CEP131* cDNA was amplified using PCR and inserted into pEGFP-C1 (Clontech) to generate a mammalian expression plasmid for GFP-tagged CEP131. Plasmids pGEX2TK-P-GST-14-3-3ɛ and pGEX2TK-P-GST-14-3-3ζ were a gift from Alistair Cook, Gurdon Institute, University of Cambridge, UK. Plasmids pEGFP-14-3-3ɛ and pEGFP-14-3-3ζ were a gift from Max Douglas, London Research Institute, UK. The S47A, S47E, S78A, S78E and S731A mutations in CEP131 were introduced by site-directed mutagenesis using KOD DNA polymerase (Millipore) according to the manufacturer's instructions. For generation of inducible GFP-CEP131 expression plasmids, GFP-CEP131 (WT, S47A/S78A, S47E7S78E) was cloned into pcDNA5/FRT/TO (Life Technologies) using the BamHI and EcoRV restriction sites. For generation of FLAG-Difopein and FLAG-Difopein (Lys) expression plasmids, the published Difopein peptide sequence^22^ was codon-optimized for expression in human cells and cDNA was ordered as a synthetic gene (Geneart, Thermo Fisher). These cDNA sequences were then subcloned into pFLAG-CMV2 (Sigma-Aldrich) using EcoRI and SalI restriction sites. Alternatively, for stable and Doxycycline-inducible cell lines, codon-optimized FLAG-Difopein was subcloned into pcDNA4/TO (Life Technologies) using KpnI and BamHI restriction sites. All constructs were verified by sequencing. Plasmid transfections were performed using FuGene 6 (Promega) or Lipofectamine (Life Technologies), and siRNA transfections were carried out with Lipofectamine RNAiMAX (Life Technologies) following the manufacturers' protocol. siRNA target sequences (Eurofins) used in this study were as follows:

Control (siCTRL; 5′-GGGAUACCUAGACGUUCUA-3′);

MK2 (5′-CCGAAAUCAUGAAGAGCAUTT-3′);

CEP131 (5'-CUGACAAACUUGGAGAAAUUTT-3');

CEP131 3′UTR (5′-GUUGAGAUGCCCACGGCUATT-3′); and

PCM1 (5′-GGUUUUAACUAAUUAUGGATT-3′).

### Cell culture and reagents

Human U2OS osteosarcoma cells were cultured in DMEM medium supplemented with 10% fetal bovine serum, L-glutamine, penicillin and streptomycin. For SILAC labelling, cells were cultured in media containing either L-arginine and L-lysine (Light), L-arginine [^13^C6] and L-lysine [^2^H4] (Medium) or L-arginine [^13^C6-^15^N4] and L-lysine [^13^C6-^15^N2] (Heavy; Cambridge Isotope Laboratories). All cells were cultured at 37 °C in a humidified incubator containing 5% CO_2_. To generate cell lines stably expressing FLAG-Difopein, cells were co-transfected with pcDNA4/TO-FLAG-Difopein and pcDNA6/TR (Life Technologies) in a 1:1 ratio and selected with Zeocin and Blasticidin (5 μg ml^−1^) for 14 days. Individual clones were picked and analysed for FLAG-Difopein expression using immunofluorescence (IF) and western blot (WB) analyses. To generate cell lines inducibly expressing GFP-CEP131, U2OS Flp-In T-rex cells were co-transfected with pOG44 recombinase construct (Life Technologies) and pcDNA5/FRT/TO-GFP-CEP131 (WT, S47A/S78A, S47E/S78E) at a 10:1 ratio. Cells were selected with Hygromycin B (200 μg ml^−1^) and Blasticidin (5 μg ml^−1^) for 14 days. Individual clones were picked and analysed for GFP expression using fluorescence microscopy and WB analyses. RPE1 (hTERT-immortalized human retinal pigment epithelial cells) was obtained from ATCC and maintained in DMEM/F12 medium containing 10% fetal bovine serum. Ultraviolet irradiation (50 J m^−2^) was delivered in a BS-02 irradiation chamber equipped with 254 nm bulbs (Gröbel Elektronik, Germany). Inhibitors used were p38 inhibitor SB203580 (10 μM, Cell Signaling), MK2 inhibitor PF3644022 (10 μM, Sigma) and MK2 inhibitor III (10 μM, Calbiochem). Anisomycin (1 μg ml^−1^, Sigma-Aldrich). GST-tagged 14-3-3ɛ and 14-3-3ζ were purified from *E. coli*. GST-tagged 14-3-3ɛ and 14-3-3ζ were mixed in a 1:1 ratio for use in GST-pull-down experiments. For SILAC experiments, we labelled U2OS cells with stable isotopes of lysine and arginine to obtain cultures with light (L), medium (M) and heavy (H) proteins, the differential mass of which can be conveniently discriminated by MS. These cell cultures were then exposed to ultraviolet and/or kinase inhibitors and the lysates were mixed and processed for phosphoproteomics.

### *In vitro* phosphorylation assays

GFP- or Strep-HA-tagged CEP131 was immunopurified from transfected U2OS cells lysed in high-salt EBC buffer (50 mM Tris, pH 7.5; 500 mM NaCl; 1 mM EDTA; 0.5% NP40; 1 mM dithiothreitol (DTT)). Beads were washed extensively with lysis buffer and once with kinase buffer (25 mM HEPES, pH 7.2; 25 mM MgCl; 2 mM DTT). Reactions were initiated by adding 200–450 ng recombinant MK2 (Abcam) and 25 μM ATP to each sample. To assay MK2-mediated phosphorylation, ATP was spiked with [γ-^32^P-ATP (10 μCi, Perkin Elmer). Indicated samples were supplemented with 100 ng recombinant HSP27 (Prospec) to serve as positive control for MK2 activity. Reactions were incubated for 30 min at 30 °C with gentle shaking and terminated by adding Laemmli buffer and boiling the samples at 95 °C for 10 min. Samples were then run on SDS–PAGE and vacuum-dried on a Whatman filter paper (Sigma). Relative phosphorylation was assayed by radioblotting. To assay MK2-dependent 14-3-3 binding *in vitro*, GFP-CEP131 immobilized on beads was treated with 1 μl Lambda Phosphatase (New England Biolabs) and incubated for 30 min at 30 °C before *in vitro* phosphorylation. The subsequent MK2 kinase assay was performed as above but without radioactive ATP. Following the kinase reaction, beads were re-suspended in low-salt EBC buffer (50 mM Tris, pH 7.5; 150 mM NaCl; 1 mM EDTA; 0.5% NP40; 1 mM DTT) and washed three times. Subsequently, purified GST-14-3-3 was added to each sample and incubated for 1 h at 4 °C while rotating, and then washed five times in low-salt EBC buffer and analysed by WB analysis.

### Immunochemical methods

GFP immunoprecipitations were performed with GFP-Trap agarose beads (Chromotek) and CEP131 immunoprecipitations were performed with CEP131 antibody (ab84864, Abcam) coupled to Protein A Sepharose beads (GE Healthcare). FLAG pull-down was performed using FLAG-M2 agarose (Sigma-Aldrich) and GST pull-down was performed with Glutathione Sepharose beads (GE Healthcare). All immunoprecipitations were carried out in low-salt EBC lysis buffer (150 mM NaCl; 50 mM Tris, pH 7.5; 1 mM EDTA; 0.5% NP40). Antibodies used in this study included the following: Rabbit polyclonals to Phospho-Akt substrate RXXS*/T* (9614, Cell Signalling; WB 1:1,000), CEP131 (A301-415A, Bethyl; WB: 1:1,000; ab84864, Abcam; IF: 1:300), PCM1 (A301-150A, Bethyl; WB 1:1,000, IF 1:350), SSX2IP (HPA027306, Sigma-Aldrich; WB 1:1,000, IF 1:200), MK2 (3042, Cell Signaling; WB 1:1,000), HSP27 pS82 (9709, Cell Signaling; WB 1:2,500), CHK1 pS345 (2348, Cell Signaling; WB 1:1,000), 14-3-3 pan (BML-SA483, Enzo Life Sciences; WB 1:1,000), GST (sc459, Santa Cruz; WB 1:1,000), Pericentrin (ab4448, Abcam; IF 1:1,000) and CEP290 (ab84870, Abcam; WB 1:1,000, IF 1:100), p38 MAPK (9212S, Cell Signaling, WB: 1:1,000); mouse monoclonals to GFP (sc-9996, Santa Cruz; WB 1:1,000 and 11814460001, Roche; WB 1:1,000), γ-tubulin (T5326, Sigma-Aldrich; IF 1:250) and FLAG-M2 (F1804, Sigma-Aldrich; WB 1:1,000, IF 1:500), Phospho-p38 MAPK (Thr180/Tyr182; 9216S, Cell Signaling, WB: 1:1,000); goat polyclonal to MCM6 (sc9843, Santa Cruz; WB 1:1,000).

### IF staining and microscopy

For IF staining of CS and γ-tubulin, cells were fixed in ice-cold 1:1 methanol/acetone mixture and incubated with primary antibodies diluted in DMEM for 1 h at room temperature. Following staining with secondary antibodies (Alexa Fluor 488, 568 and 647; Life Technologies) for 30 min, coverslips were mounted with Vectashield mounting medium (Vector Laboratories) containing nuclear stain 4,6-diamidino-2-phenylindole. For fixation of IF samples without γ-tubulin staining, cells were fixed in 4% formaldehyde, permeabilized with PBS containing 0.2% Triton X-100 for 5 min and immunostained as above. Images were acquired with an LSM 780 confocal microscope (Carl Zeiss Microimaging Inc.) mounted on a Zeiss-Axiovert 100 M equipped with a Plan-Apochromat × 40/1.3 oil immersion objective, or a Leica DMI6000B wide-field microscope (Leica Microsystems) equipped with an HC Plan-Apochromatic × 63/1.4 oil immersion objective. Image acquisition and analysis were carried out with the ZEN2010 and LAS X software, respectively. Presence/absence of CS was determined by assessing individual cells for a cluster of PCM1, CEP131, CEP290 or SSX2IP in direct vicinity of the centrosome in both axial (*x, y*) and lateral (*z*) directions. For mitotic live cell imaging, cells grown on eight-well microscopy slides (Ibidi) were cultured in DMEM-based medium for 2 days before imaging. Culture medium was changed to L-15 (Life Technologies) supplemented with 10% fetal bovine serum (Hyclone) and Penicillin/Streptomycin (Life Technologies) immediately before imaging. Slides were mounted on a Delta Vision Elite microscope (GE Healthcare) and cells were imaged for 16 h in 10-min intervals using a × 40/1.35 WD 0.10 dry objective. Seven Z-Stacks with intervals of 1.5 μm were taken using differential interference contrast and a GFP filter set (32% intensity, 0.1-s exposure time). All data analyses were performed using the SoftWoRx software (GE Healthcare).

### MS analysis

Phosphorylated peptides were enriched by conventional methods^42^. Peptide fractions were analysed on a quadrupole Orbitrap mass spectrometer (Q Exactive or Q Exactive Plus, Thermo Scientific) equipped with a UHPLC system (EASY-nLC 1000, Thermo Scientific)^43^,^44^. Peptide samples were loaded on C18 reversed phase columns (15 cm in length, 75 μm inner diameter and 1.9 μm bead size) and eluted with a linear gradient from 8 to 40% acetonitrile containing 0.1% formic acid for 2 h. The mass spectrometer was operated in a data-dependent mode, automatically switching between MS and MS^2^ acquisition. Survey full-scan MS spectra (*m*/*z* 300–1,700) were acquired in the Orbitrap. The 10 most intense ions were sequentially isolated and fragmented with higher-energy C-trap dissociation^45^. An ion selection threshold of 5,000 was used. Peptides with unassigned charge states, as well as with charge states less than +2 were excluded from fragmentation. Fragment spectra were acquired in the Orbitrap mass analyser.

### Peptide identification

Raw data files were analysed using MaxQuant^46^. Parent ion and MS^2^ spectra were searched against a database containing 88,473 human protein sequences obtained from the UniProtKB released in December 2013 using the Andromeda search engine^47^. Spectra were searched with a mass tolerance of 6 p.p.m. in the MS mode, 20 p.p.m. in the higher-energy C-trap dissociation MS^2^ mode, strict trypsin specificity and allowing up to three miscleavages. Cysteine carbamidomethylation was searched as a fixed modification, whereas protein N-terminal acetylation, methionine oxidation and phosphorylation of serine, threonine and tyrosine were searched as variable modifications. Site localization probabilities were determined by MaxQuant using the PTM scoring algorithm^46^. The data set was filtered based on posterior error probability to arrive at a false discovery rate of below 1% estimated using a target-decoy approach^48^.

## Additional information

**How to cite this article:** Tollenaere, M. A. X. *et al*. p38- and MK2-dependent signalling promotes stress-induced centriolar satellite remodelling via 14-3-3-dependent sequestration of CEP131/AZI1. *Nat. Commun.* 6:10075 doi: 10.1038/ncomms10075 (2015).

## Supplementary Material

Supplementary Figures Supplementary Figures 1-11

Supplementary Data 1 UV-, p38-, and MK2-regulated phosphorylation sites on centriolar satellite proteins.

Supplementary Movie 1 Time-lapse of U2OS: Flp-In T-Rex GFP-CEP131 WT cells. Cells were grown in CO2-independent medium on glass-bottom dishes and imaged for GFP fluorescence over the course of 10 min. The movie is speeded up 60x compared to real time.

Supplementary Movie 2 Time-lapse of U2OS: Flp-In T-Rex GFP-CEP131 (S47A/S78A) cells. Cells were grown in CO2-independent medium on glass-bottom dishes and imaged for GFP fluorescence over the course of 10 min. The movie is speeded up 60x compared to real time.
