# Supplementary material for: p38- and MK2-dependent signalling promotes stress-induced centriolar satellite remodelling via 14-3-3-dependent sequestration of CEP131/AZI1
Source: Nat Commun. 2015 Nov 30;6:10075. doi: 10.1038/ncomms10075 (PMC4674683; doi:10.1038/ncomms10075)
Supplement: Supplementary Figures — 1-11 [file ncomms10075-s1.pdf]

Tollenaere et. al. Supplementary Figure 1

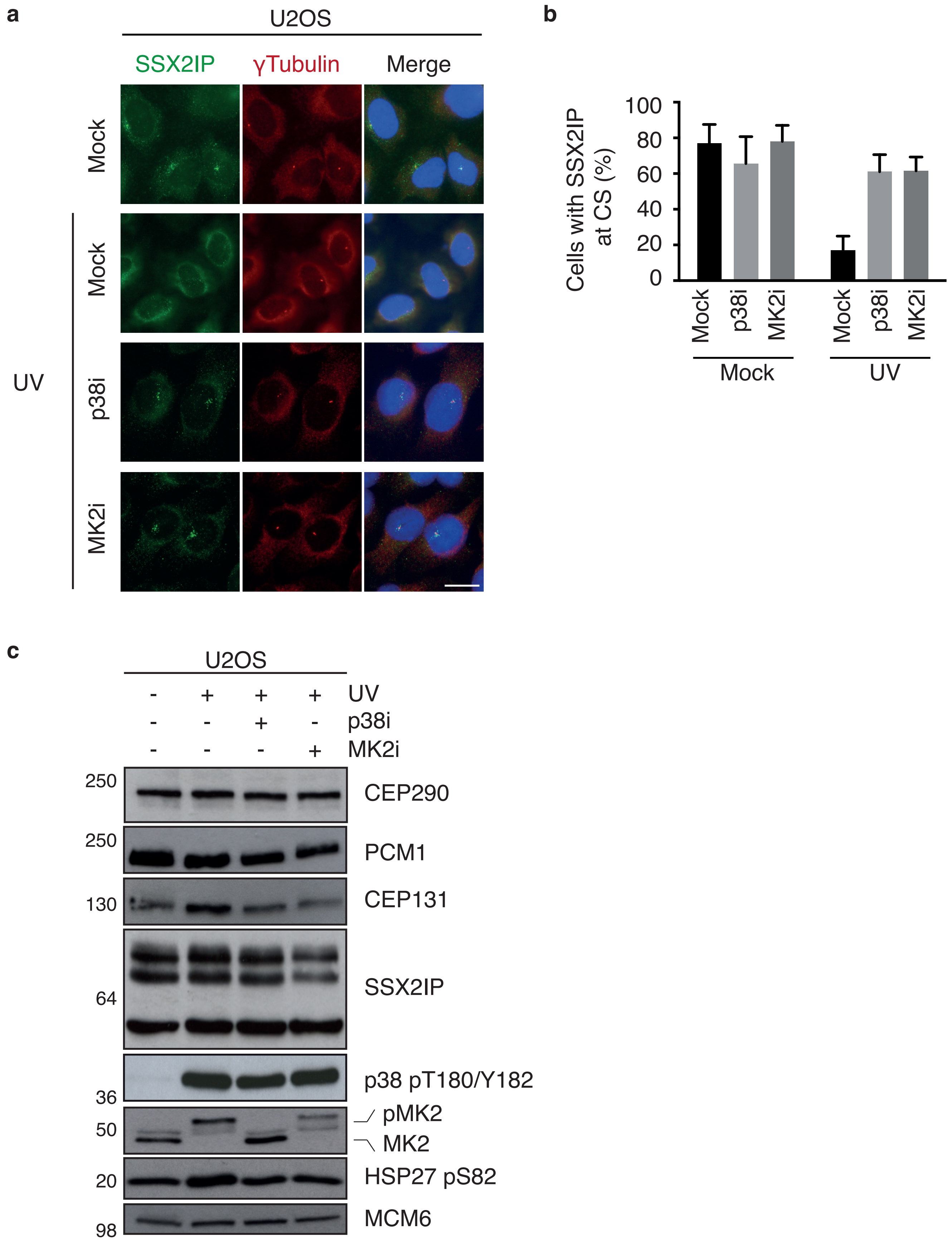

Supplementary Figure 1

Centriolar satellite dissolution after UV-irradiation in U2OS cells.

- **a.** U2OS cells were incubated with inhibitors against p38 (p38i) or MK2 (MK2i) for 1 h and exposed to UV-irradiation as indicated. Cells were fixed 1 h later and co-immunostained with SSX2IP and γ-tubulin antibodies. Scale bar, 10 μm.
- **b.** Quantification of SSX2IP localization to CS in cells treated as in (a). At least 100 cells were scored per condition. Results (mean±SD) from three independent experiments are shown. P-values were calculated from a one-tailed student's t-test.
- **c.** Lysates from cells in a) were analyzed by immunoblotting with the indicated antibodies.

Tollenaere et. al. Supplementary Figure 2

a

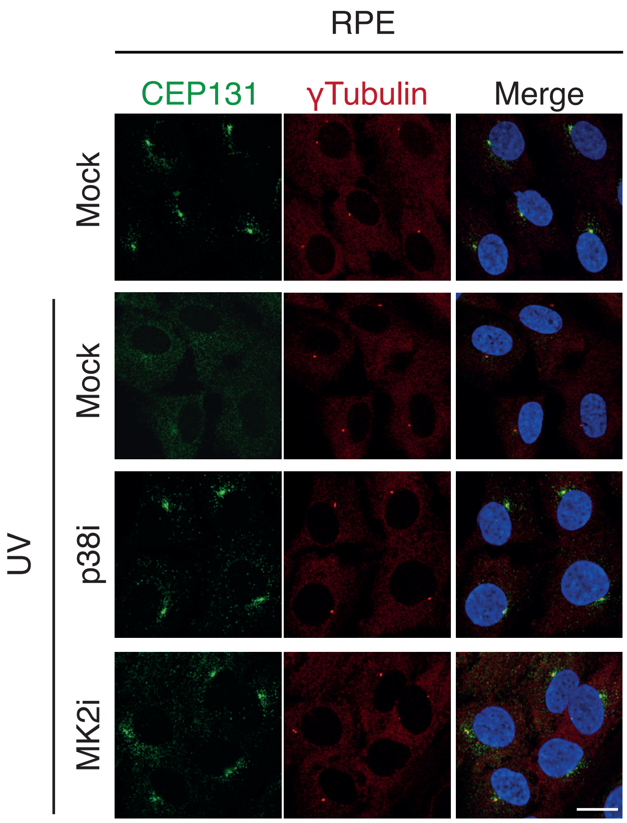

b

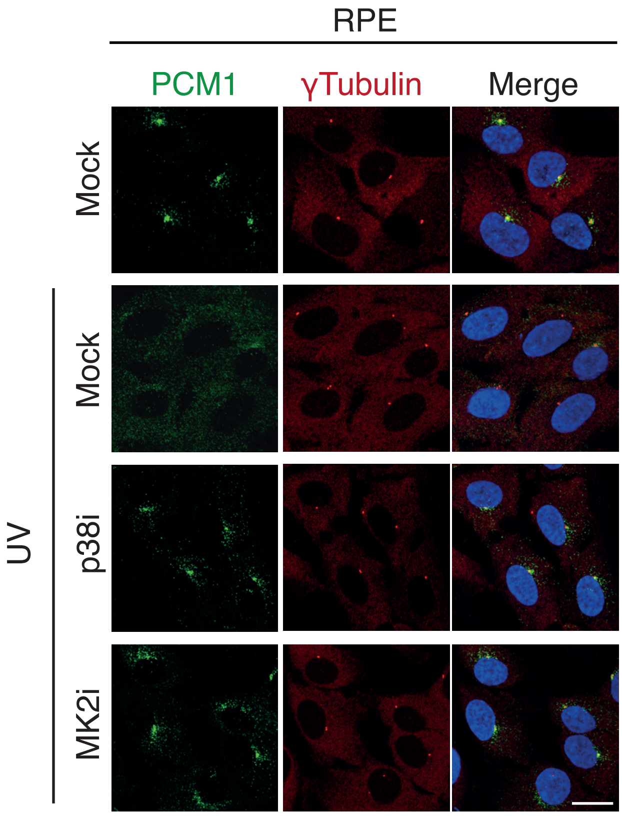

Supplementary Figure 2

Centriolar satellite dissolution after UV-irradiation in RPE cells.

- **a.** Immortalized human retinal pigment epithelial cells (RPE) were treated with p38 or MK2 inhibitors for 1 hour and fixed an additional hour after UV-irradiation. Cells were analyzed by co-immunostaining with antibodies against CEP131 and γ-tubulin.
- **b.** RPE cells were treated as in (a) and co-immunostained against PCM1 and γ-tubulin. All scale bars, 10 μm.

Tollenaere et. al. Supplementary Figure 3

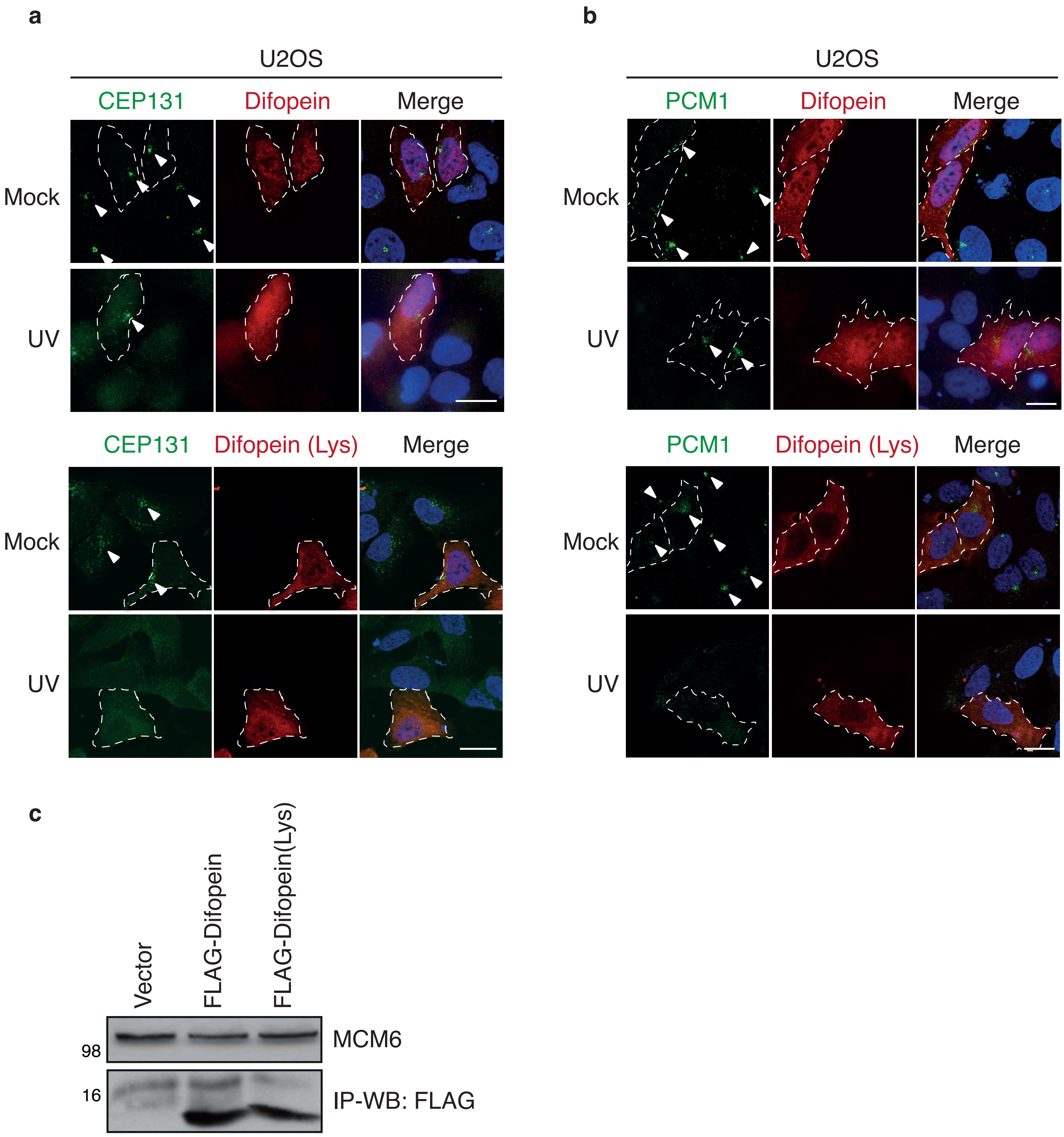

Supplementary Figure 3

Difopein prevents stress-induced centriolar satellite collapse.

- **a.** U2OS cells were transiently transfected with FLAG-Difopein or FLAG-Difopein (Lys) and exposed to UV-irradiation as indicated. Cells were fixed 1 hour after UV and immunostained with antibodies against CEP131 and FLAG. Dotted white lines highlight FLAG-difopein expressing cells and white arrowheads mark CS.
- **b.** Cells were transfected and treated as in (a), except that they were immunostained with antibodies against PCM1 and FLAG. Dotted white lines highlight FLAG-Difopein expressing cells and white arrowheads mark CS. All scale bars, 10 μm.
- **c.** U2OS cells were transfected with FLAG-Difopein or FLAG-Difopein (Lys) constructs. Input lysates and FLAG immunoprecipitates (IP) were analyzed by immunoblotting with the indicated antibodies.

Tollenaere et. al. Supplementary Figure 4

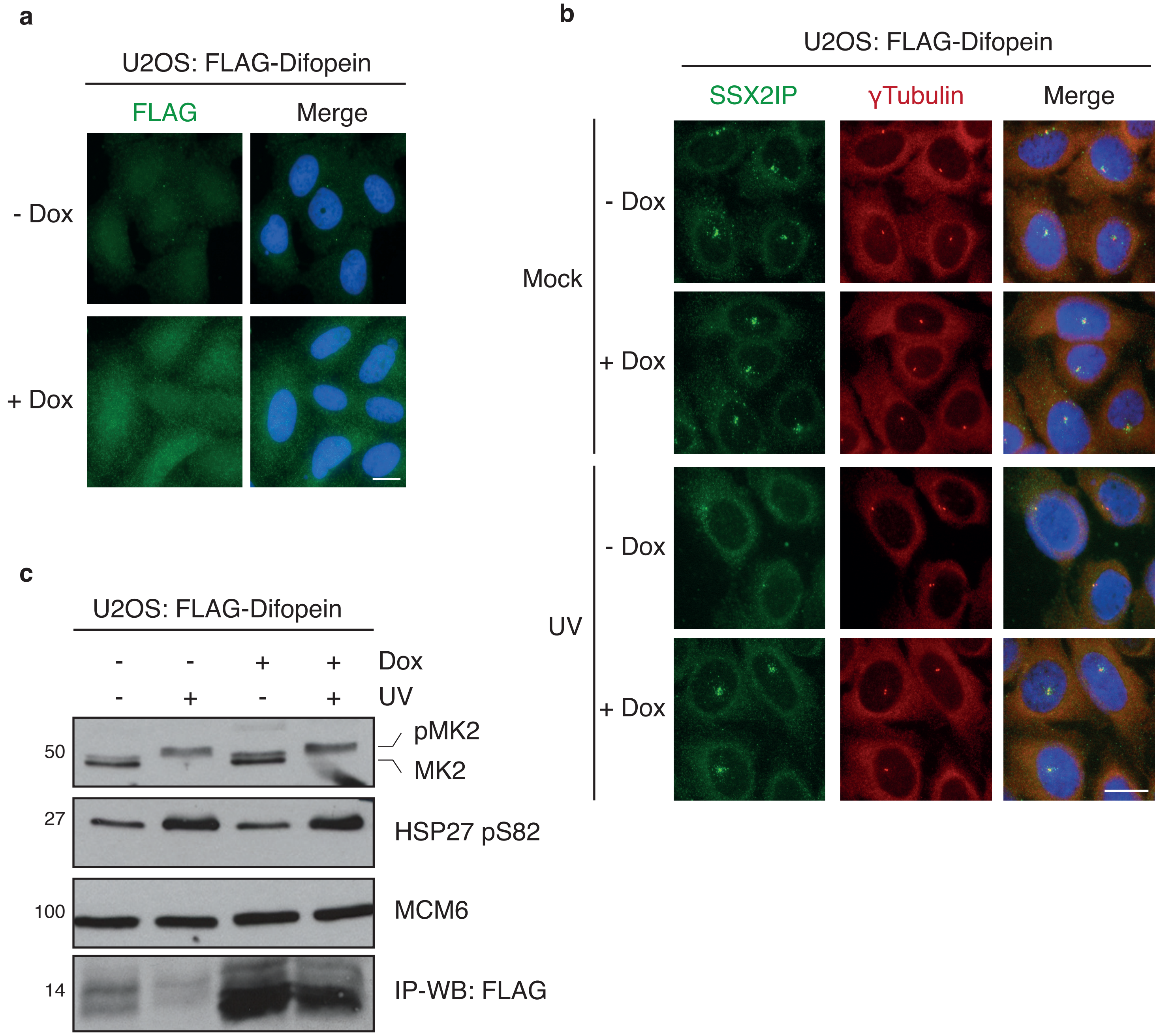

Supplementary Figure 4

Characterization of inducible U2OS: FLAG-Difopein cell line

- **a.** U2OS: FLAG-Difopein cells were induced with 2 μg ml^{-1} doxycycline (Dox) for 24 hours, fixed and immunostained with antibodies against FLAG-tag.
- **b.** Cells were treated as in (a), fixed 1 h after UV and co-immunostained with antibodies against SSX2IP and γ-tubulin. Scale bars, 10 μm.
- **c.** U2OS: FLAG-Difopein cells were treated as in (a) and (b) and assayed for p38 and MK2 activation by immunoblotting lysates with the indicated antibodies.

Tollenaere et. al. Supplementary Figure 5

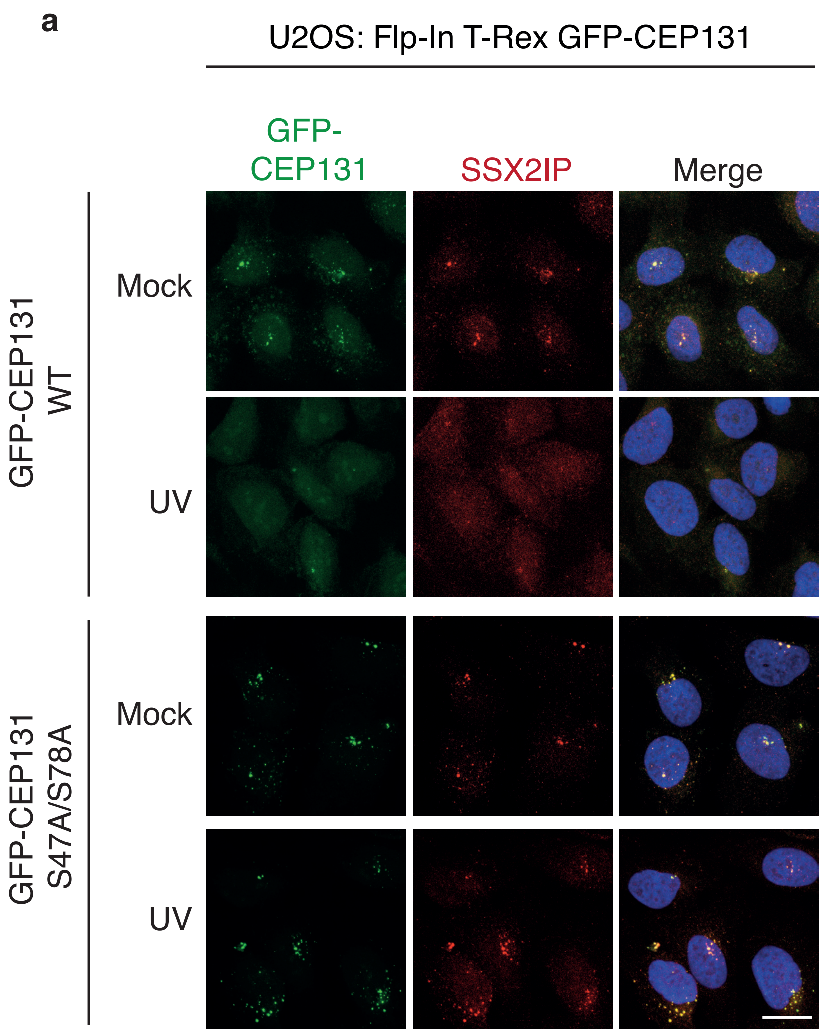

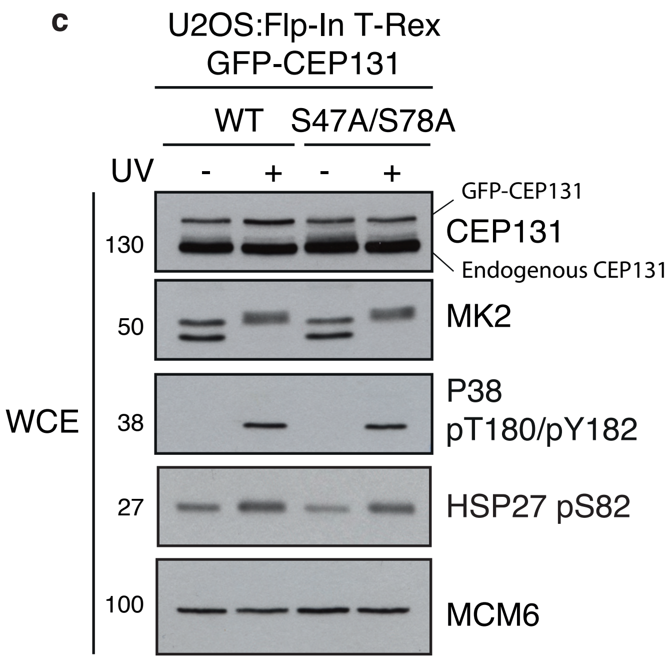

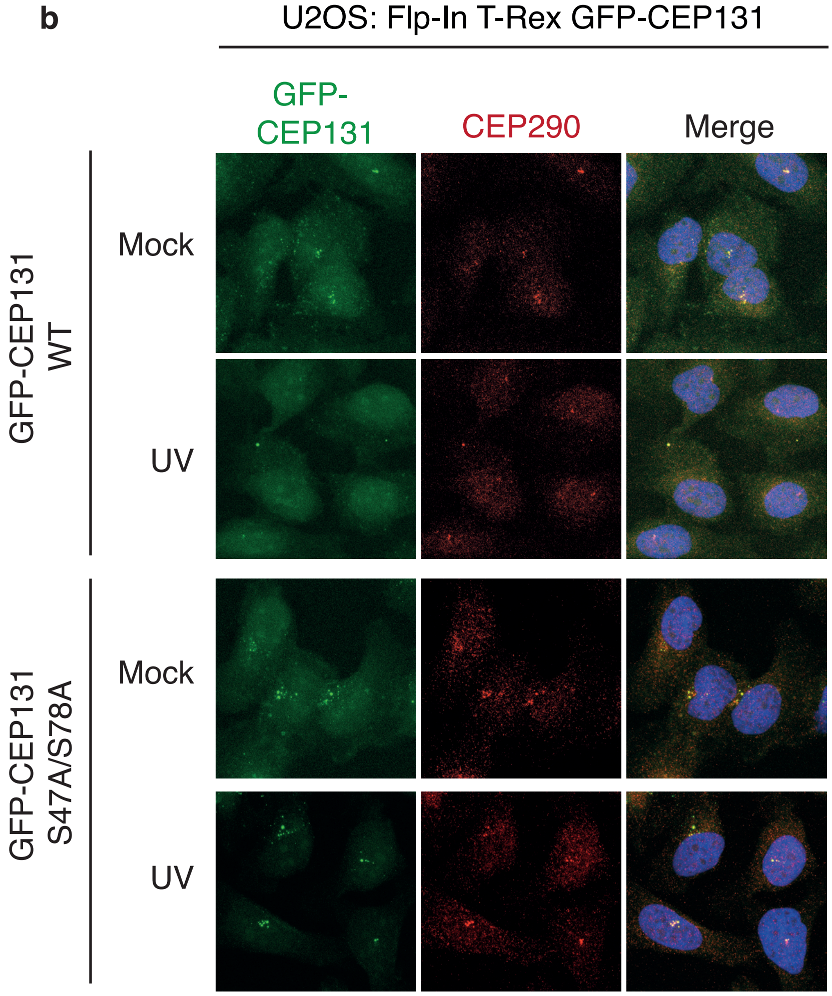

Supplementary Figure 5

UV-induced displacement of SSX2IP and CEP290 from centriolar satellites requires CEP131 phosphorylation.

- **a.** U2OS: Flp-In T-Rex GFP-CEP131 WT and mutant (S47A/S78A) cells were exposed to UV-irradiation as indicated. Cells were fixed and immunostained with antibodies against SSX2IP.
- **b.** As in (a), except that cells were immunostained with antibodies against CEP290. All scale bars, 10 µm.
- **c.** Cells were treated as in (a) and lysates were analyzed by immunoblotting with the indicated antibodies.

Tollenaere et. al. Supplementary Figure 6

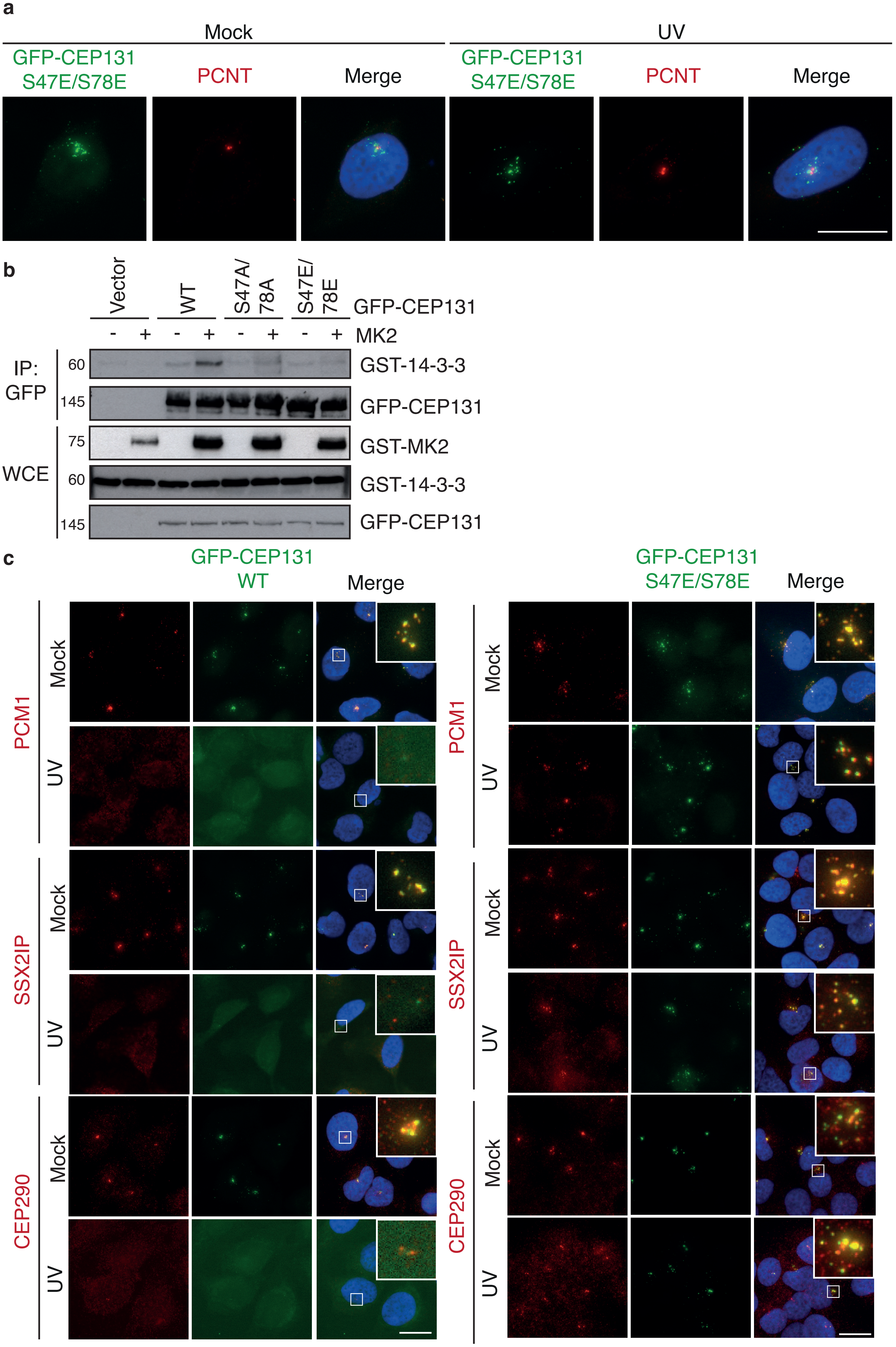

Supplementary Figure 6

"Phosphomimicking" CEP131 is refractory to 14-3-3 binding and CS remodeling.

- **a.** U2OS:FlpIn/T-Rex GFP-CEP131 S47E/S78E cells were treated with UV-irradiation, fixed and stained with antibodies against pericentrin.
- **b.** Wild type (WT) and mutant versions of GFP-CEP131 were expressed in U2OS cells, isolated on GFP-Trap agarose and treated with lambda phosphatase prior to *in vitro* phosphorylation by recombinant GST-MK2. After washing, beads were incubated with GST-14-3-3 for 1 h and CEP131-14-3-3 binding was analyzed by immunoblotting with the indicated antibodies.
- **c.** U2OS:FlpIn/T-Rex GFP-CEP131 WT and S47E/S78E cells were exposed to UV-irradiation, fixed and co-immunostained with the indicated antibodies. All scale bars, 10 μm. Inserts show magnified regions of CS.

Tollenaere et. al. Supplementary Figure 7

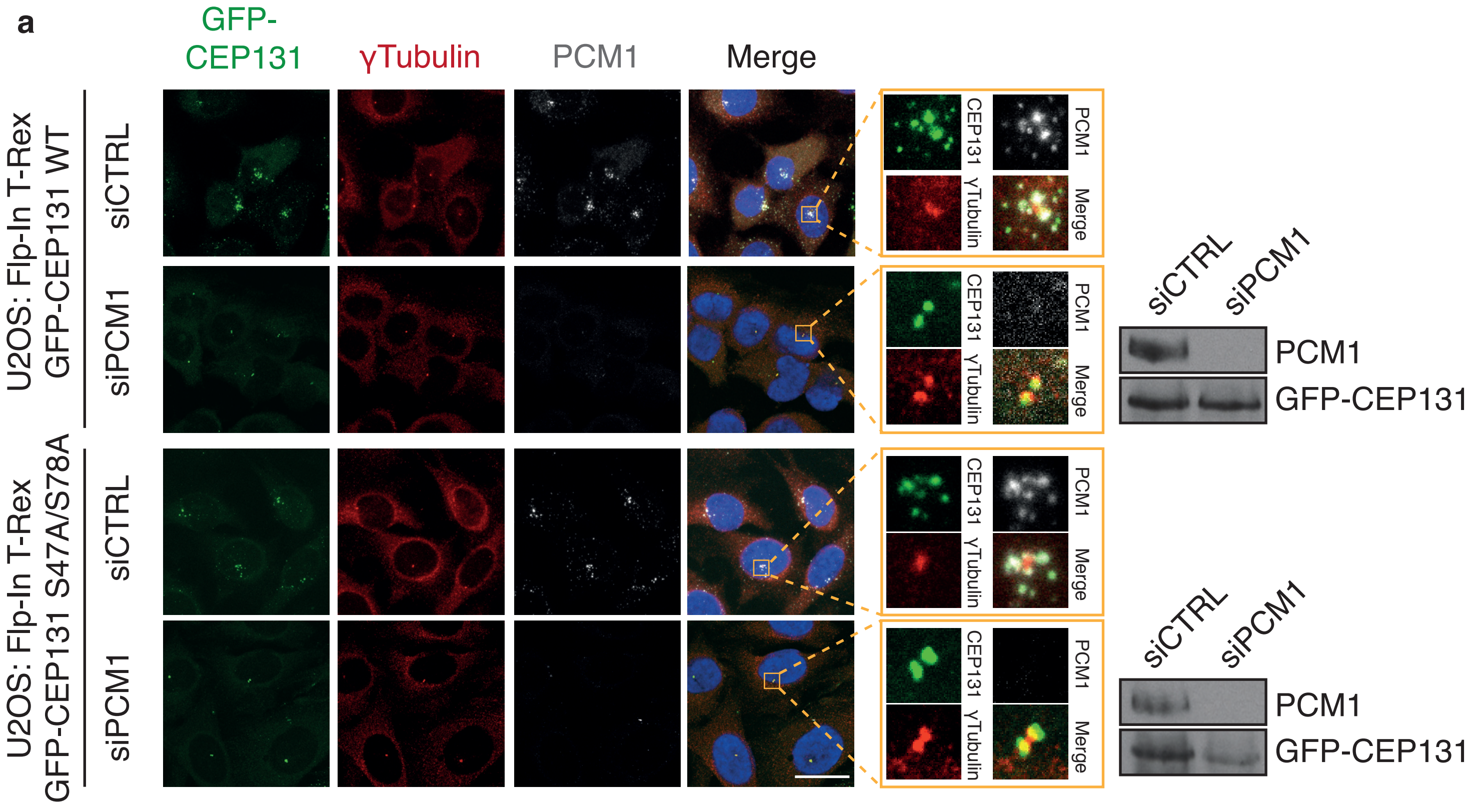

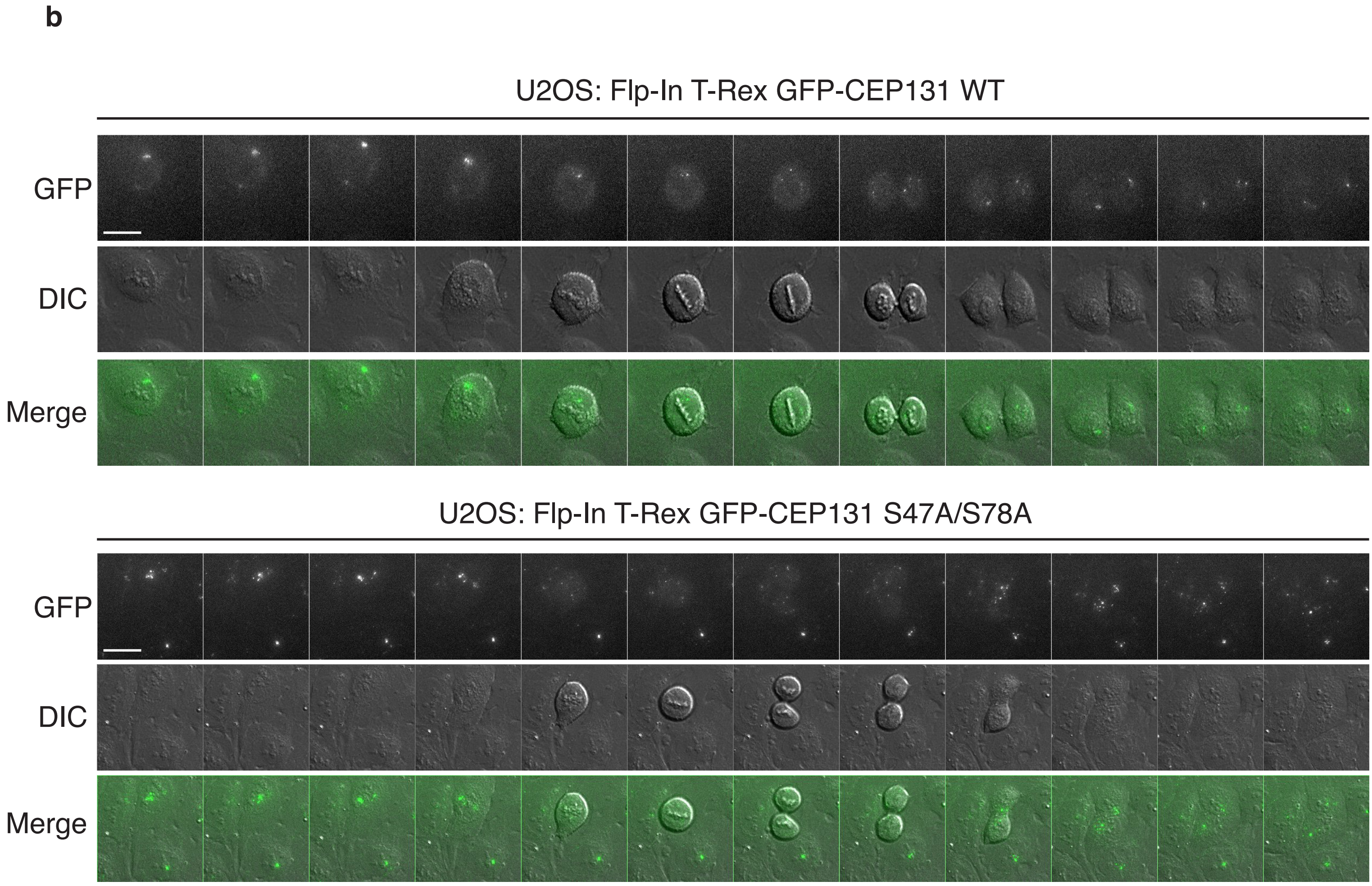

Supplementary Figure 7

Dynamic behavior of centriolar satellites in U2OS: Flp-In T-Rex GFP-CEP131 cell lines.

- **a.** U2OS:FlpIn/T-Rex GFP-CEP131 WT and mutant (S47A/S78A) cells were transfected with control (CTRL) or siRNAs targeting PCM1 for 72 hours. Subsequently, cells were fixed and co-immunostained with antibodies against PCM1 and γ-tubulin. Inserts show an enlarged region around the centrosomes (marked by γ-tubulin) to distinguish between CS and centrosomal localization of GFP-CEP131. Efficient PCM1 knockdown was confirmed by immunoblotting lysates with antibodies against PCM1 and GFP.
- **b.** U2OS:Flp-In T-Rex GFP-CEP131 WT and mutant (S47A/S78A) cells were seeded in live cell imaging dishes and imaged every 10 minutes over a period of 16 hours to record GFP-CEP131 localization during mitosis. Selected images demonstrate representative cells over a period from approximately 60 minutes before anaphase until 60 minutes after (10 minutes between frames). All scale bars, 10 μm.

Tollenaere et. al. Supplementary Figure 8

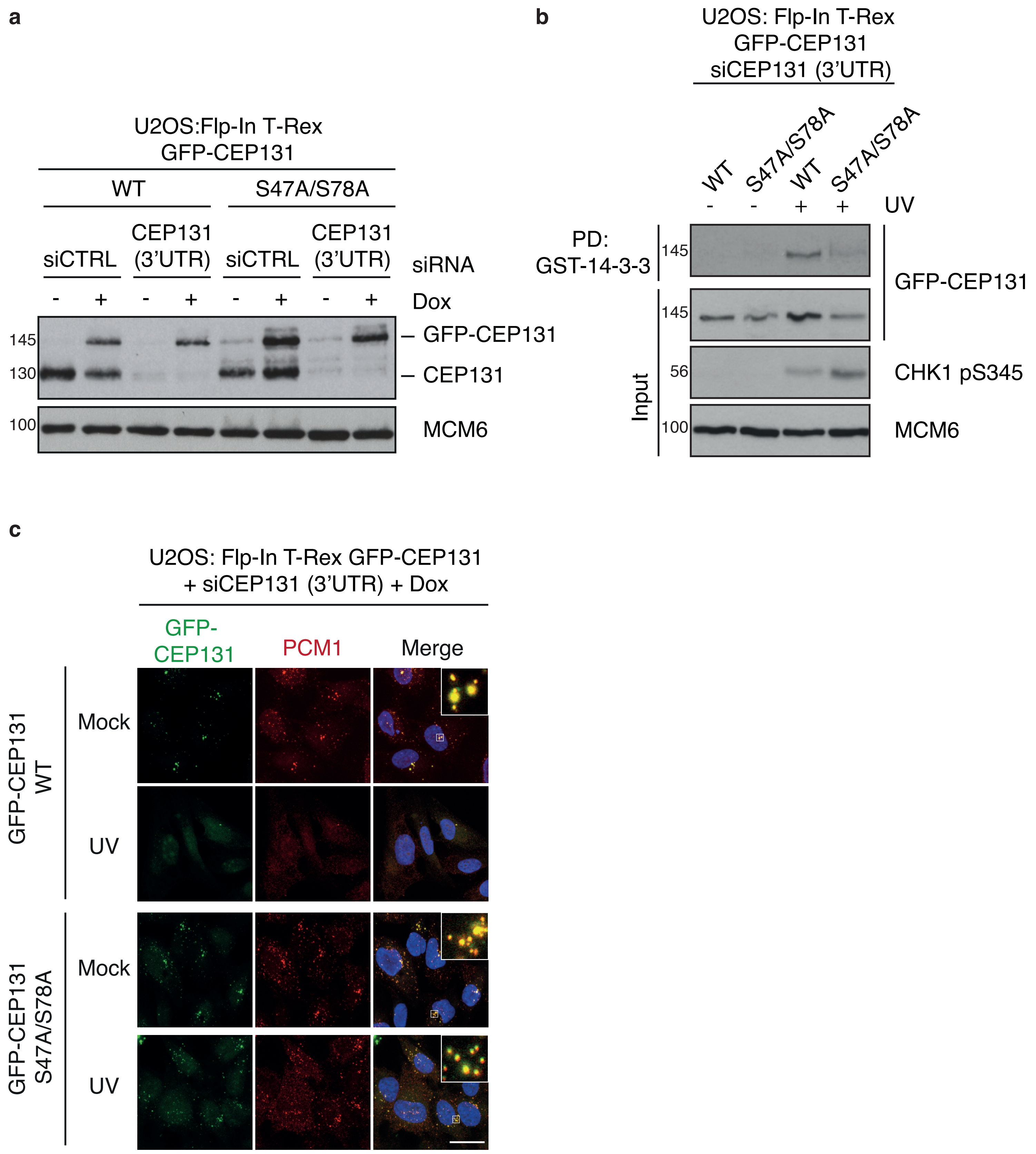

Supplementary Figure 8

CEP131 gene replacement highlights essential role for S47 and S78 in stress-induced CS remodeling.

- **a.** U2OS: Flp-In T-Rex GFP-CEP131 WT and mutant (S47A/S78A) cells were transfected with control (CTRL) or siRNA targeting a non-coding part of the CEP131 gene (siCEP131 3'UTR) and incubated in the presence of minute amounts of doxycycline (10 ng ml^{-1}) for 12 hrs. Lysates were analyzed by immunoblotting with the indicated antibodies.
- **b.** Cells were transfected with CEP131 3'UTR siRNA and induced with doxycycline as in (a). Cells were exposed to UV-irradiation, lysed after 1 h and incubated with GST-14-3-3. Input lysates and GST-bound material were analyzed by immunoblotting with the indicated antibodies.
- **c.** Cells from (b) were fixed one hour after UV-irradiation and immunostained with antibodies against PCM1. Scale bar, 10 μm. Inserts show magnified regions of CS.

Tollenaere et. al. Supplementary Figure 9

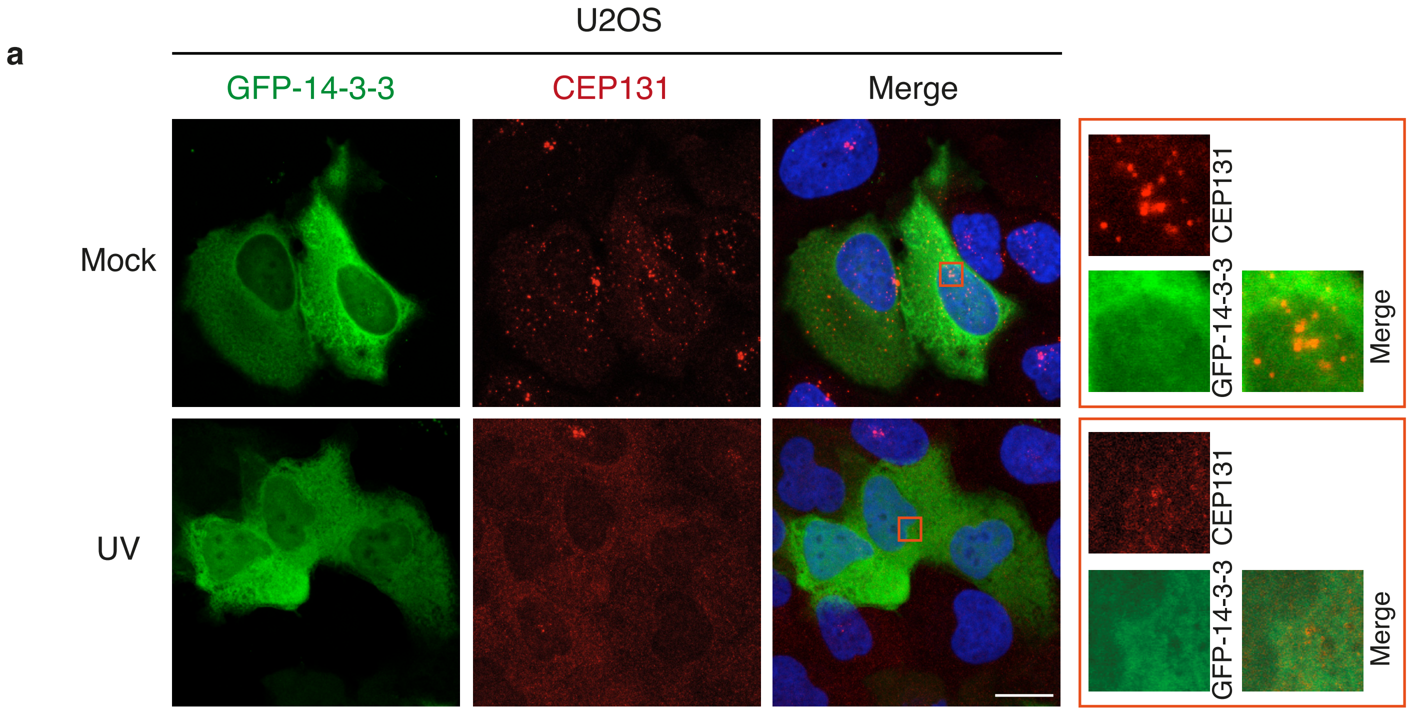

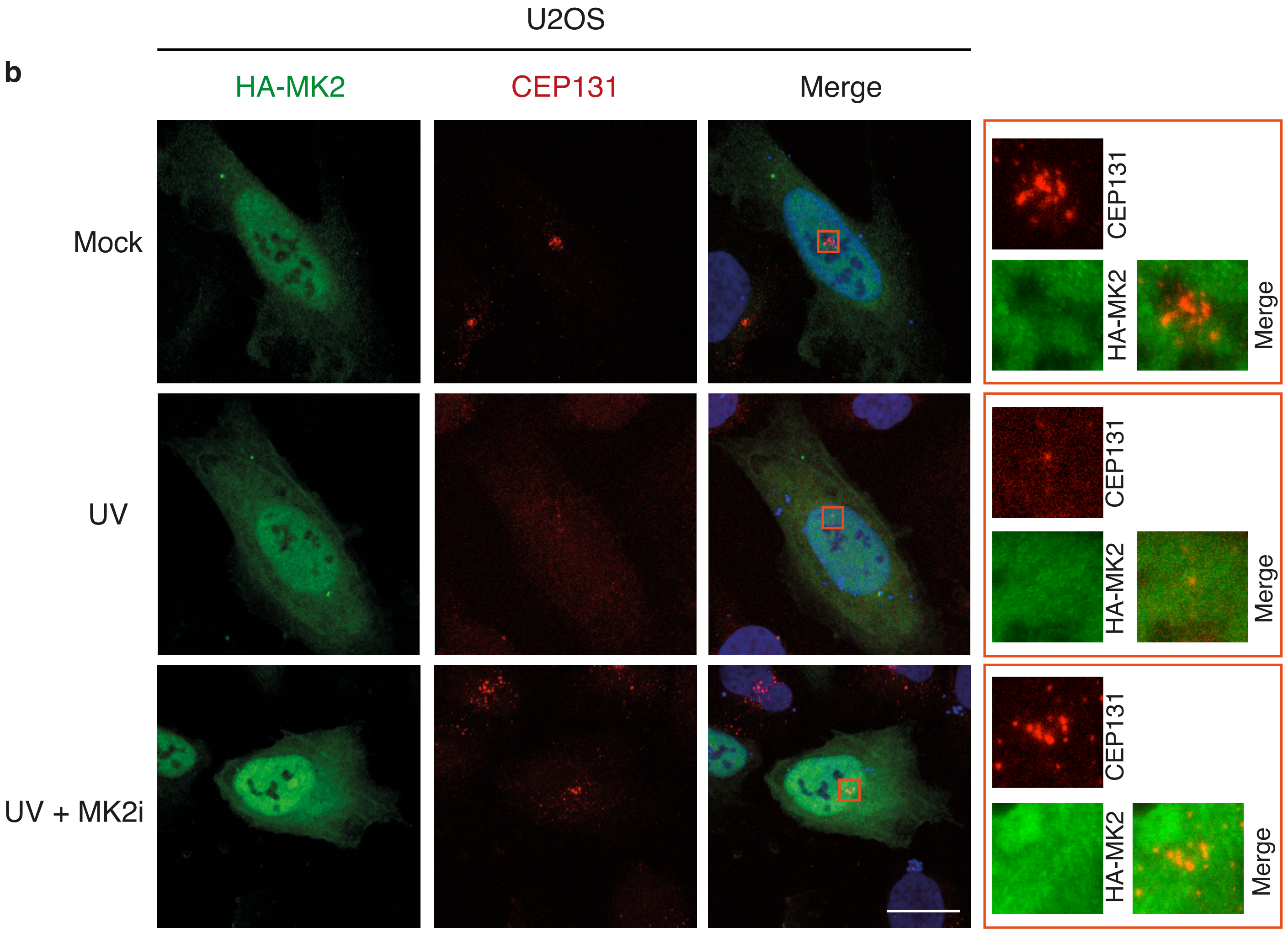

Supplementary Figure 9

14-3-3 and MK2 do not physically associate with CS.

a. U2OS cells were transfected with GFP-tagged 14-3-3 and exposed to UV-irradiation as indicated. After 1 h, cells were fixed and stained with antibodies against CEP131.

b. U2OS cells were transfected with HA-tagged MK2, pre-treated with MK2 inhibitor for 1 h, and exposed to UV-irradiation as indicated. After 1 h, cells were fixed and stained with antibodies against CEP131. Inserts show magnified regions of CS. Scale bars, 10 μm.

Tollenaere et. al. Supplementary Figure 10

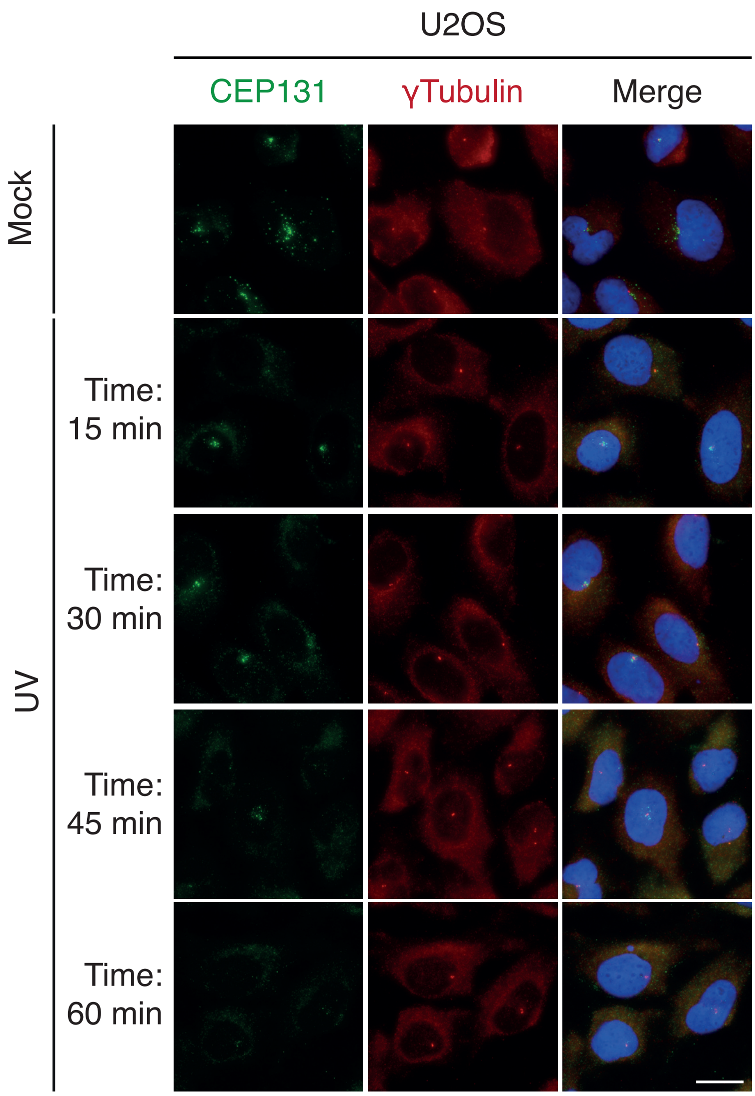

Supplementary Figure 10

CS disperse gradually after UV-irradiation.

a. U2OS cells were exposed to UV-irradiation, fixed at the indicated timepoints. and co-immunostained with CEP131 and γ-tubulin antibodies. Scale bar, 10 μm.

Tollenaere et. al. Supplementary Figure 11 (1 of 6)

Figure 2c

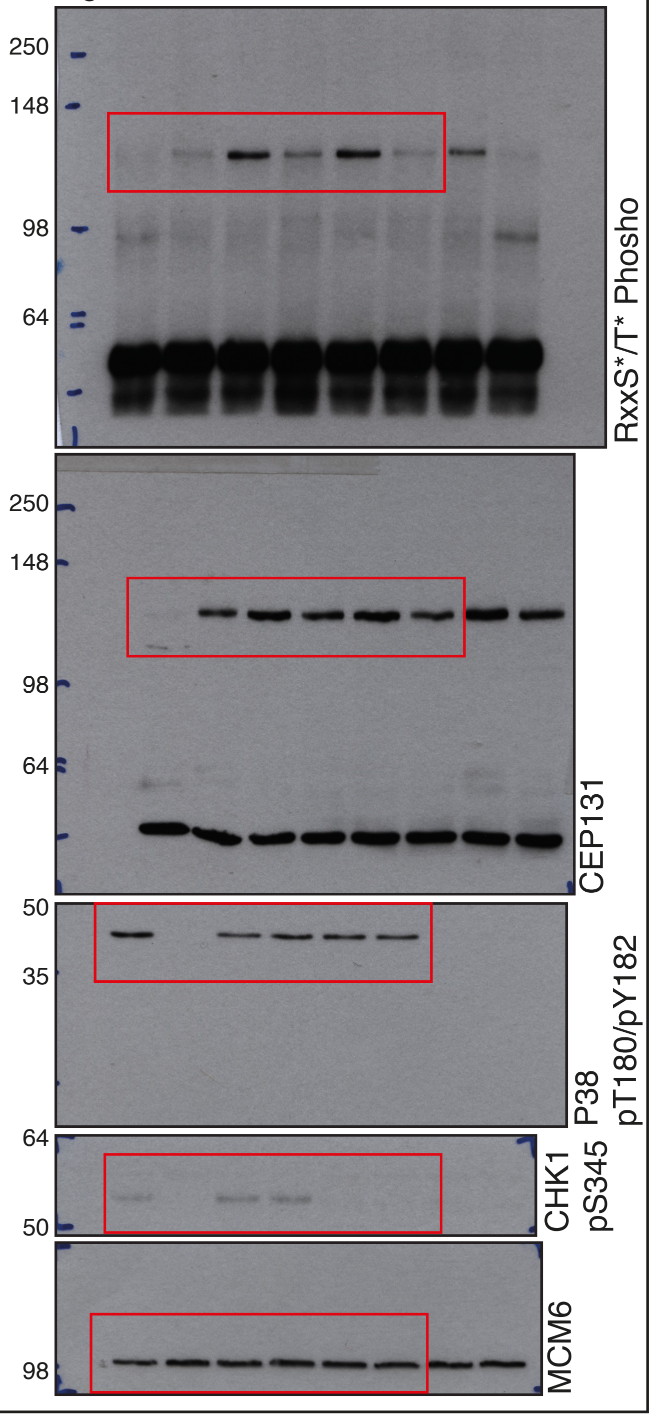

Figure 2f

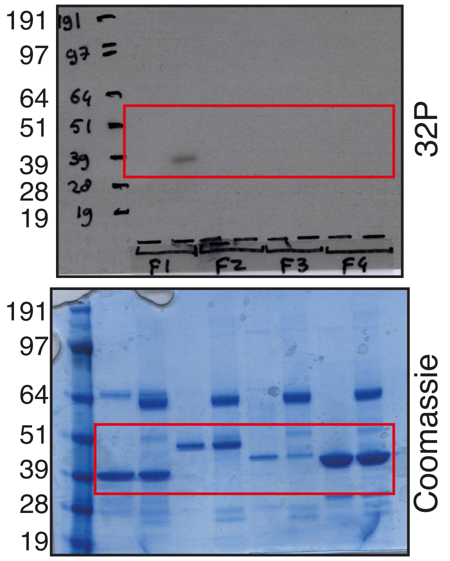

Figure 2g

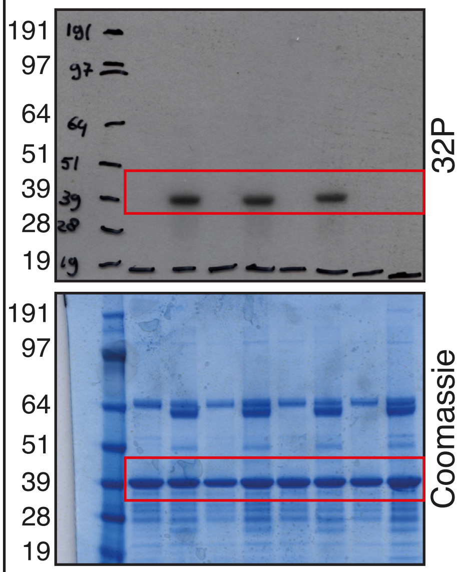

Tollenaere et. al. Supplementary Figure 11 (2 of 6)

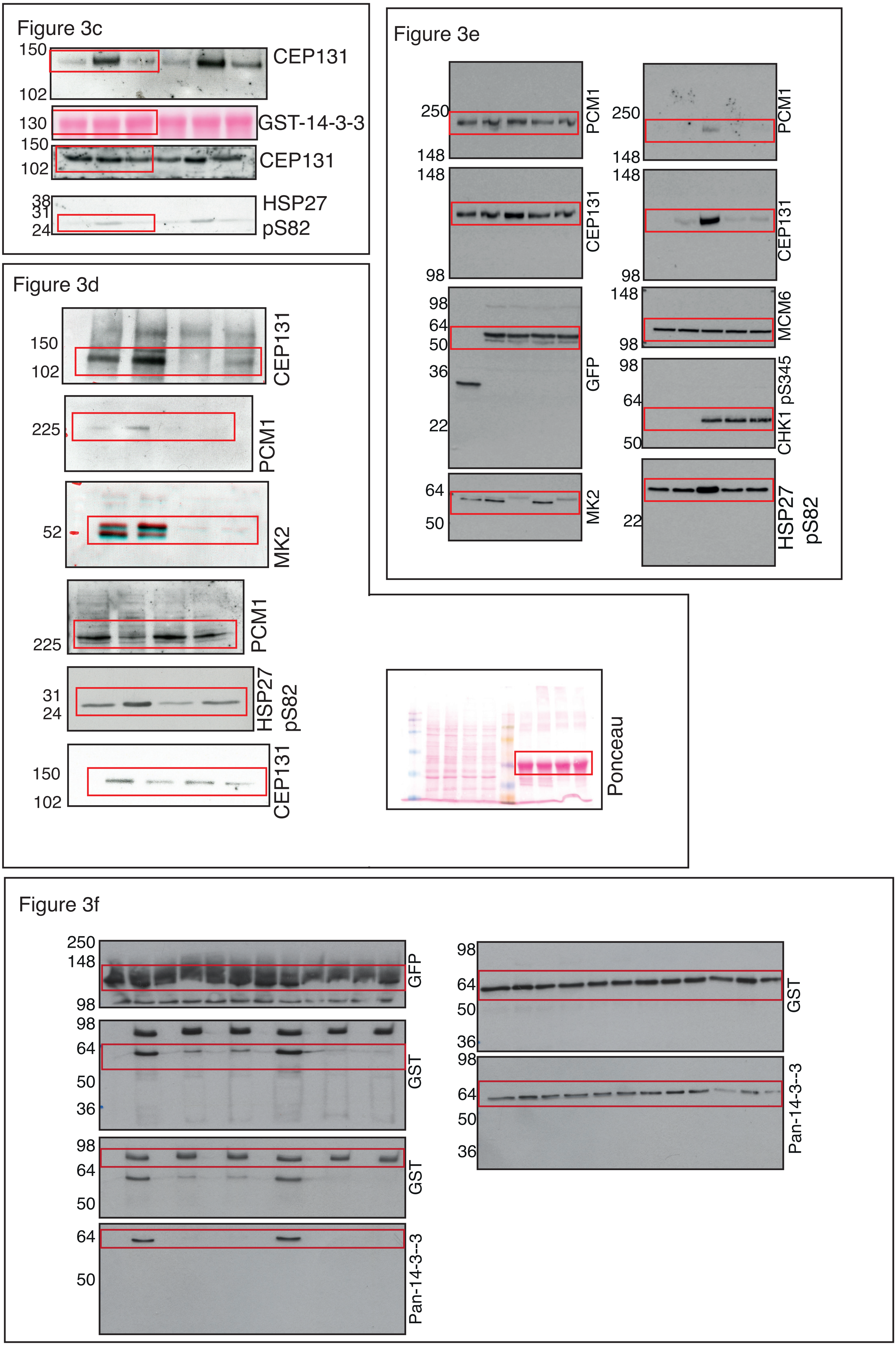

Tollenaere et. al. Supplementary Figure 11 (3 of 6)

Figure 4b

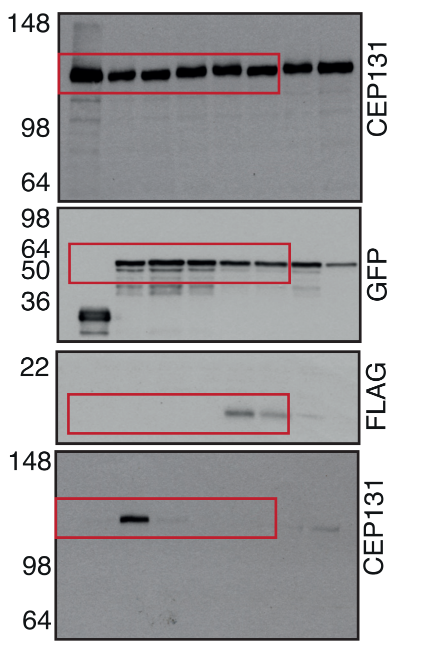

Figure 5a

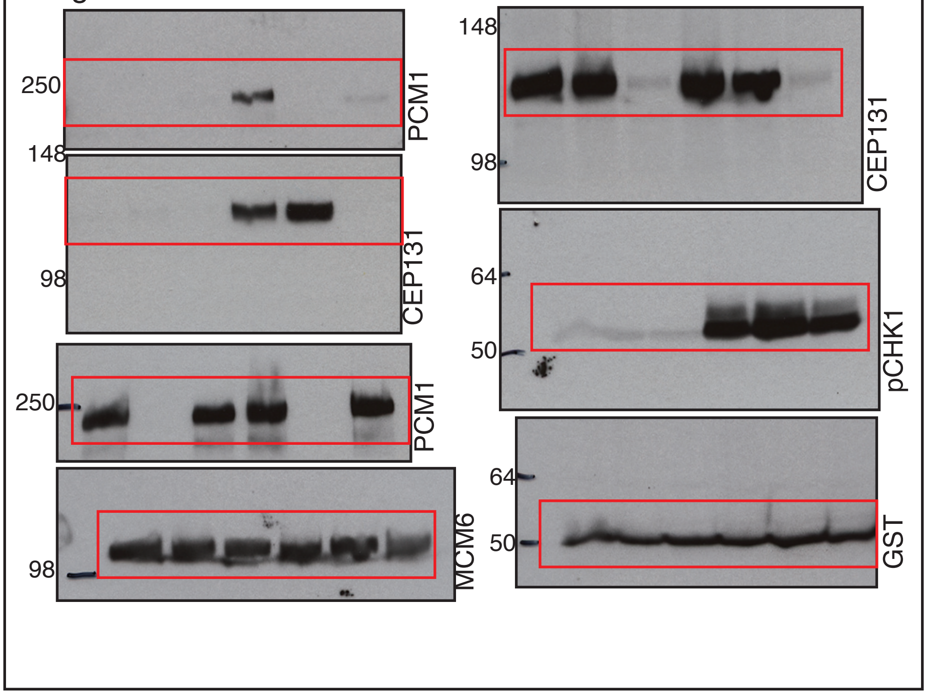

Figure 4e

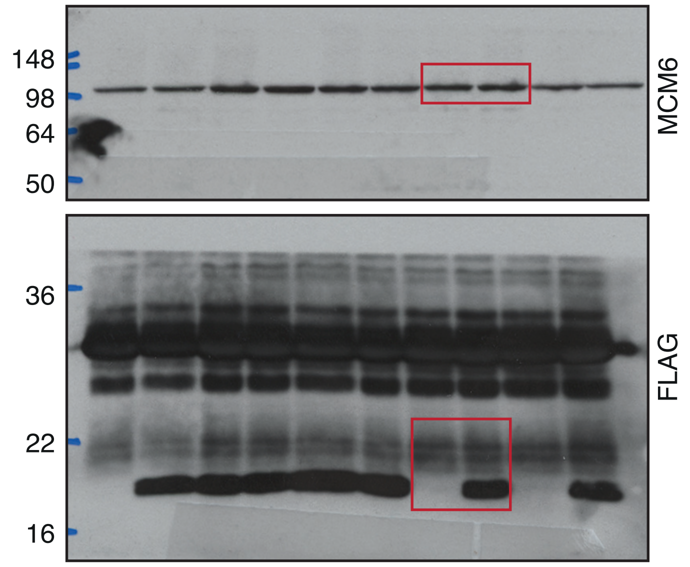

Figure 5c

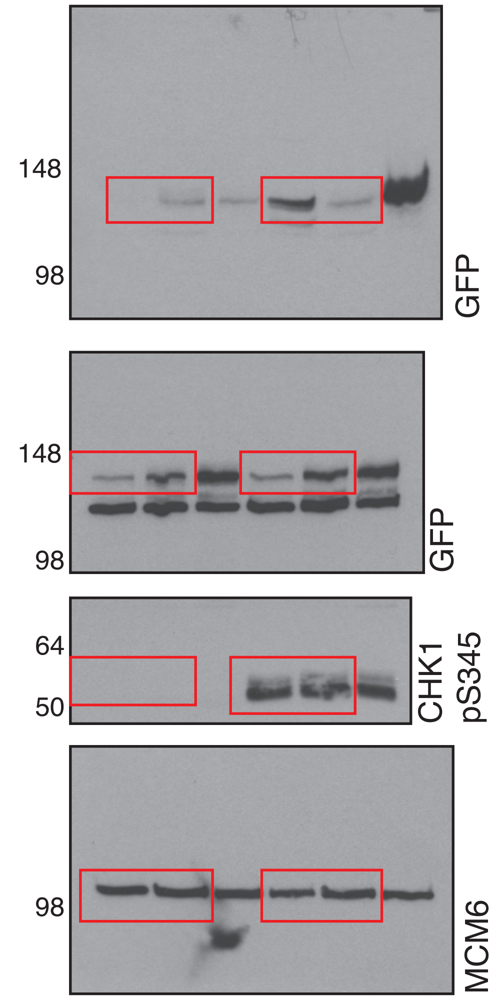

Figure 4f

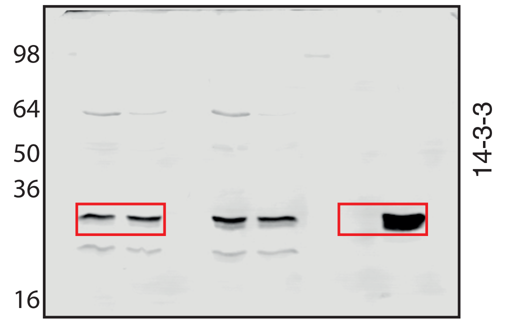

Figure 5b

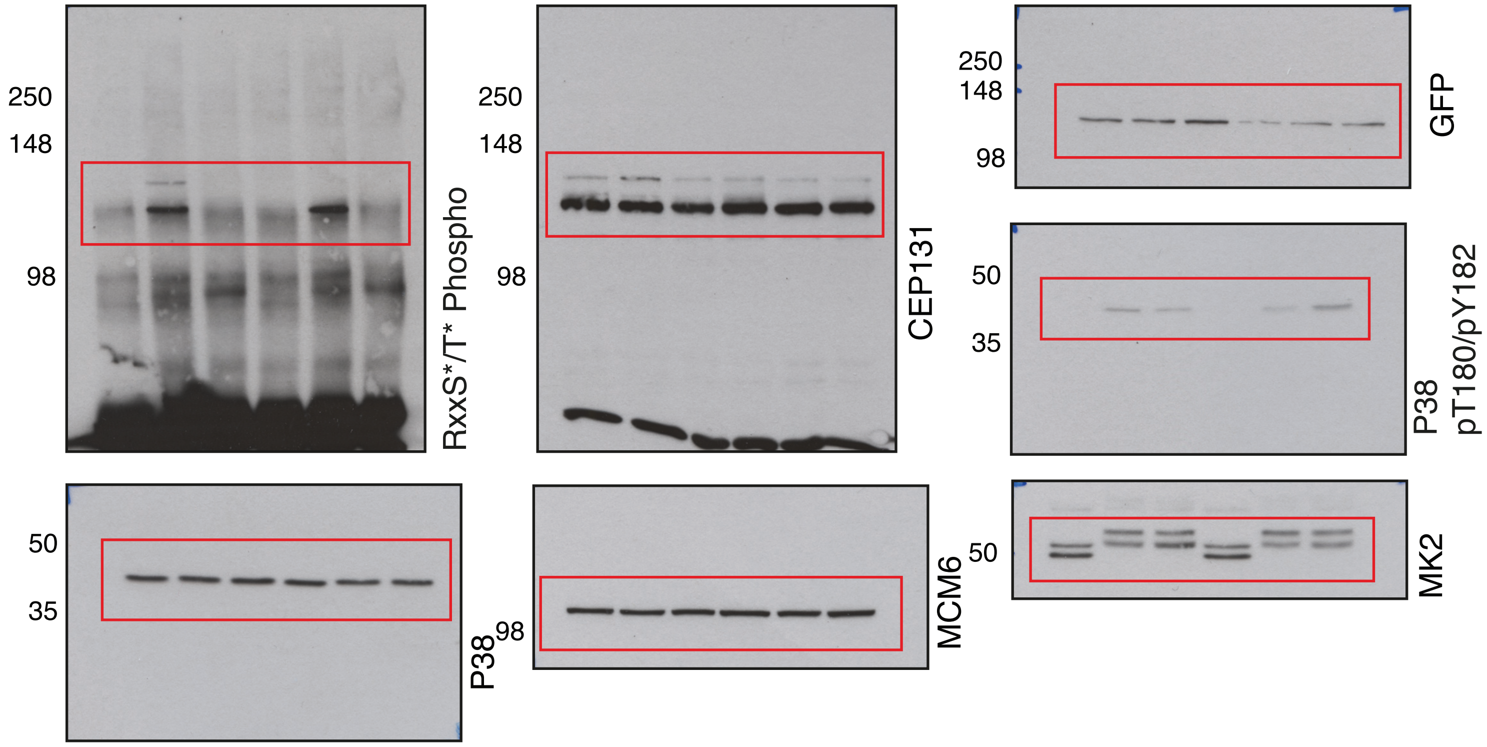

Figure 6a

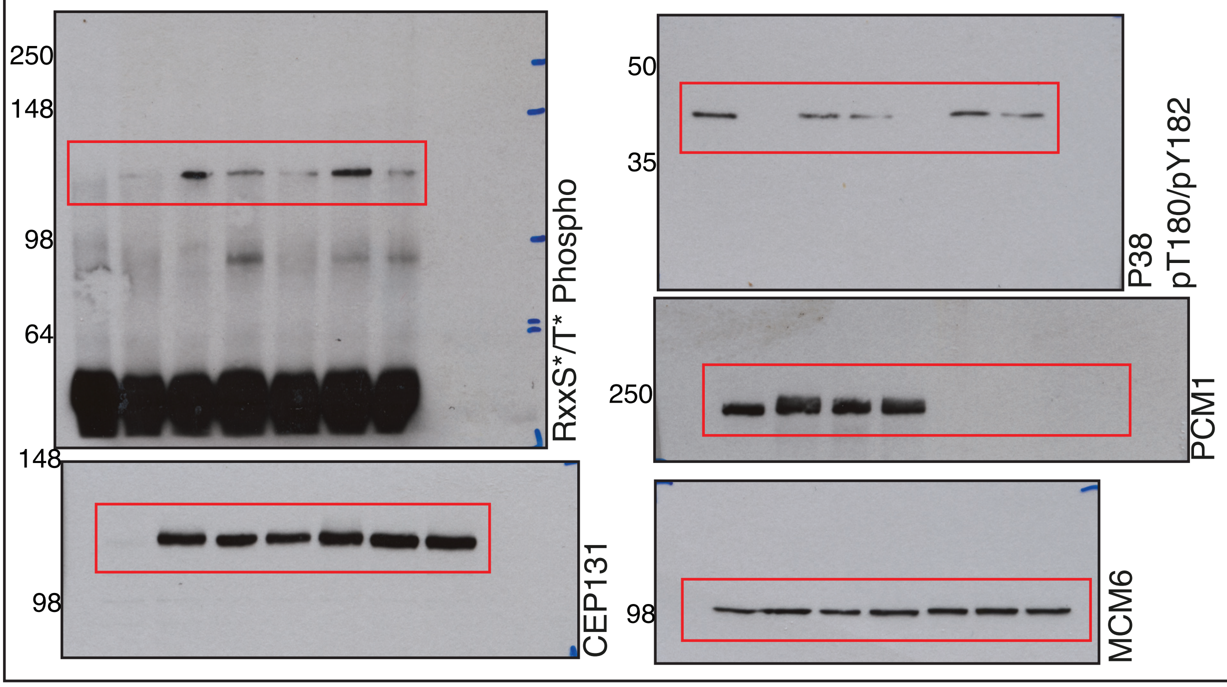

Figure 6c

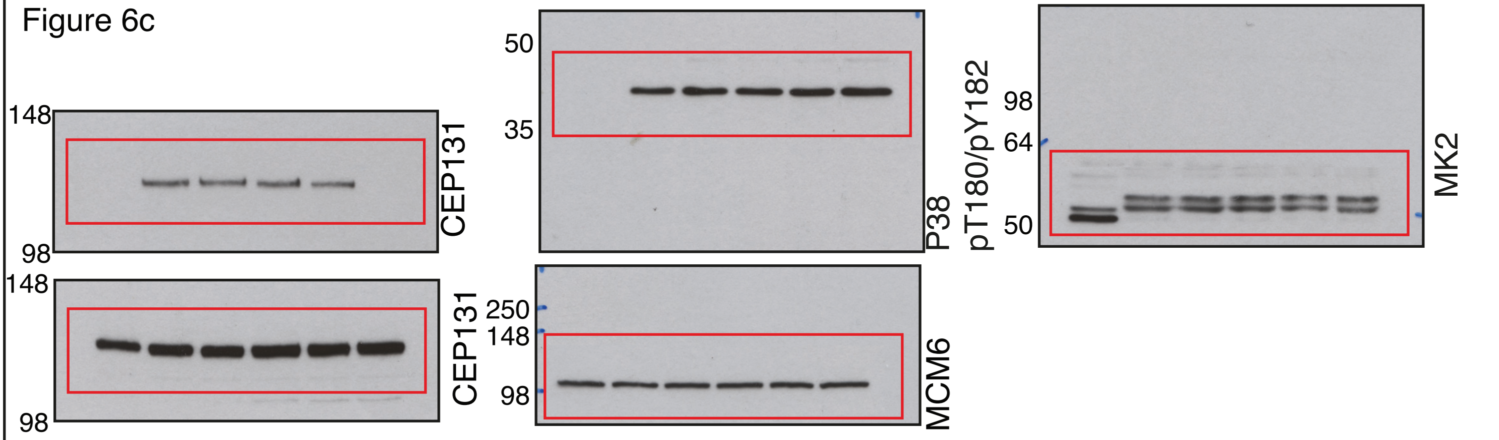

Tollenaere et. al. Supplementary Figure 11 (5 of 6)

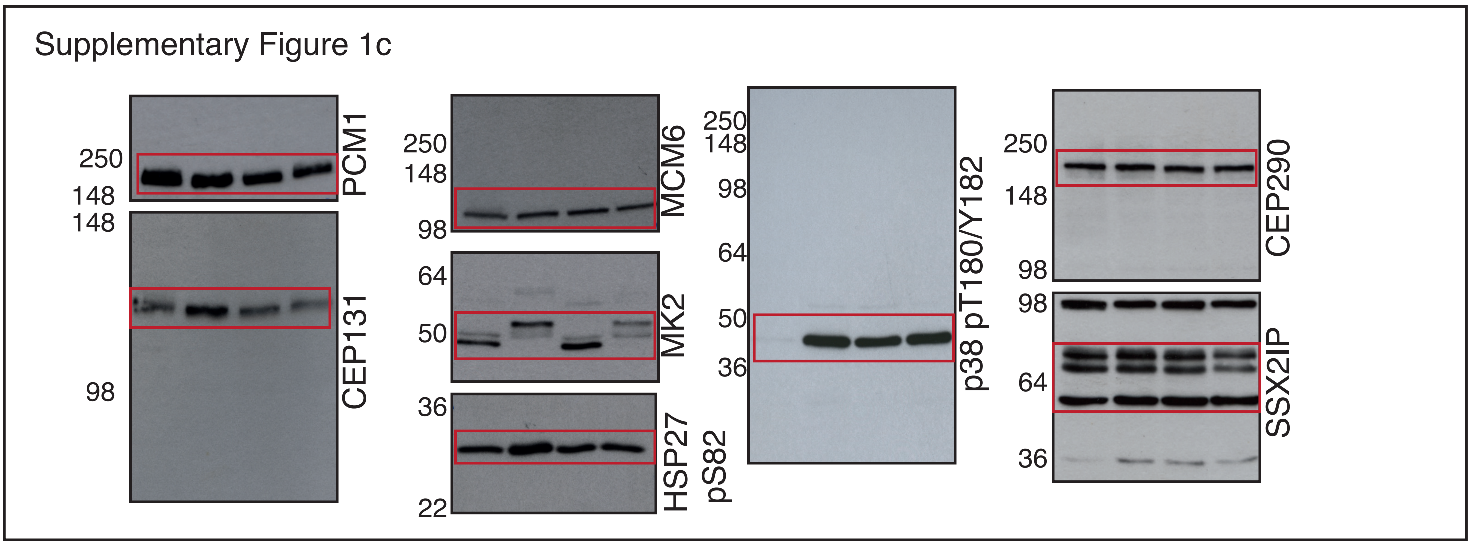

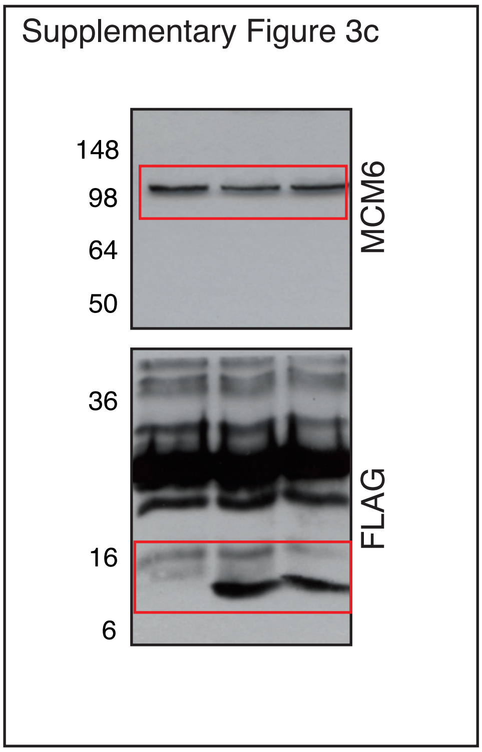

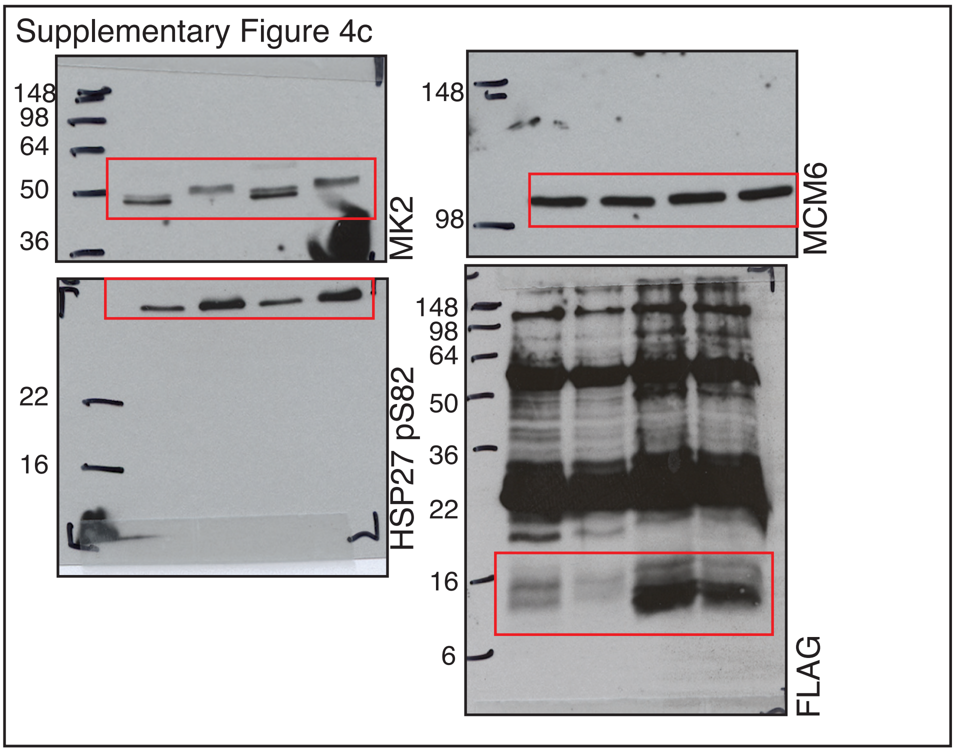

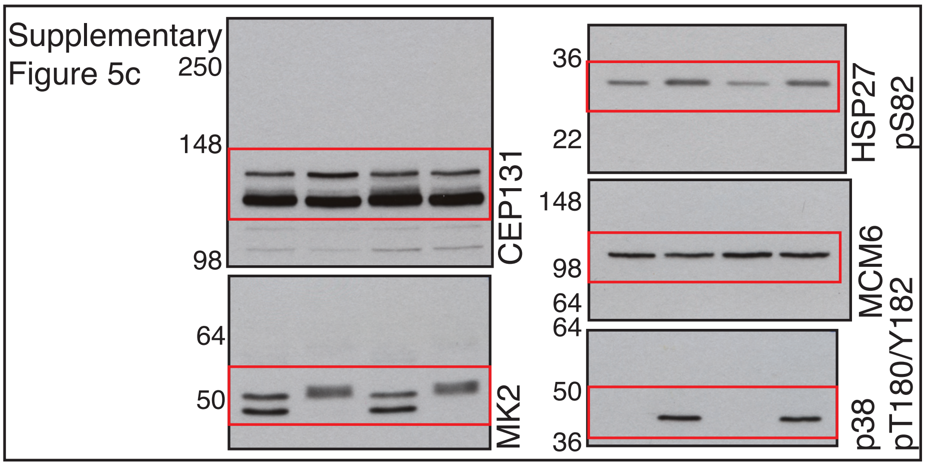

Tollenaere et. al. Supplementary Figure 11 (6 of 6)

Supplmentary Figure 6b

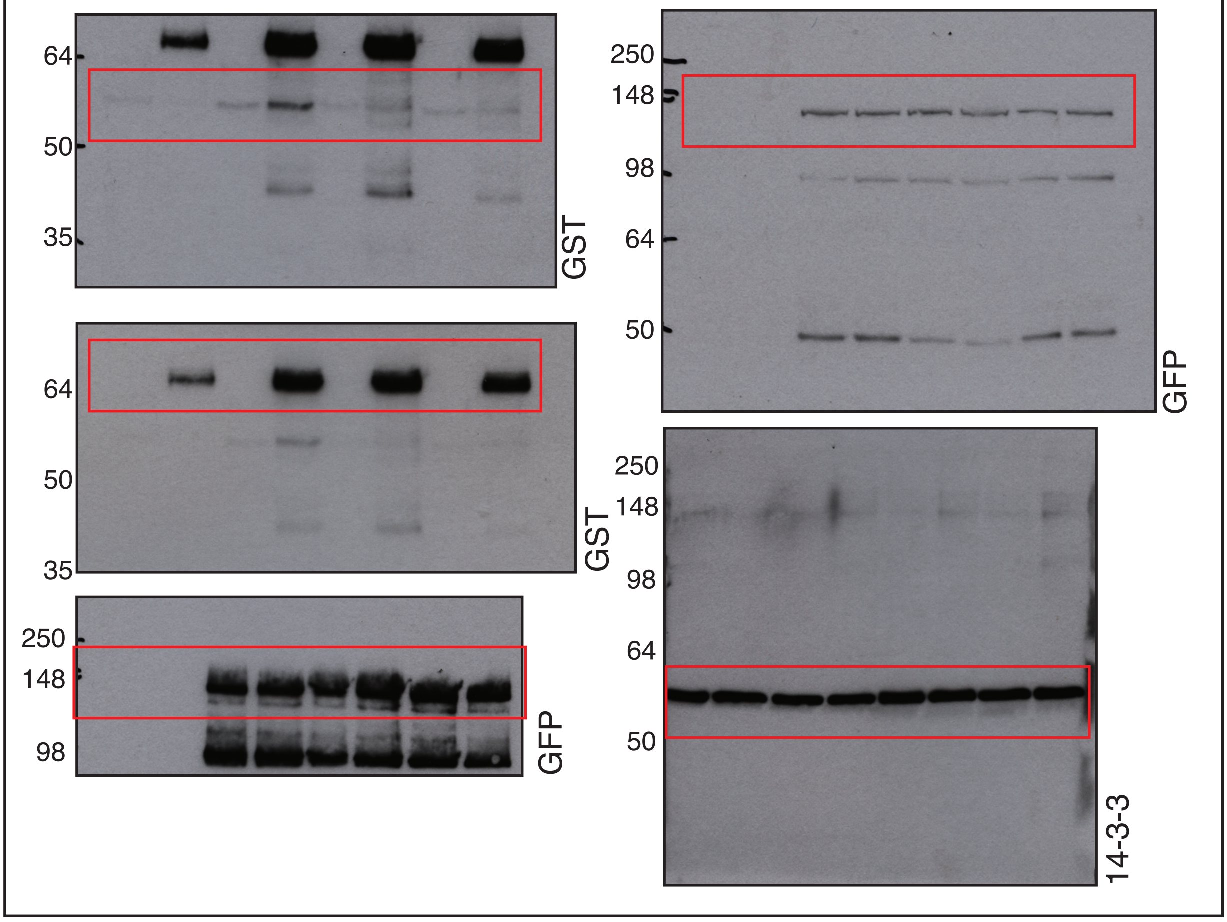

Supplementary Figure 7a

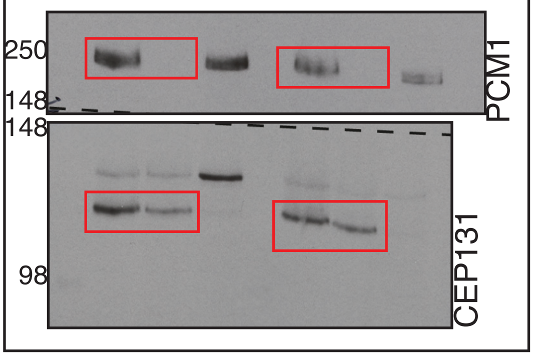

Supplementary Figure S8a

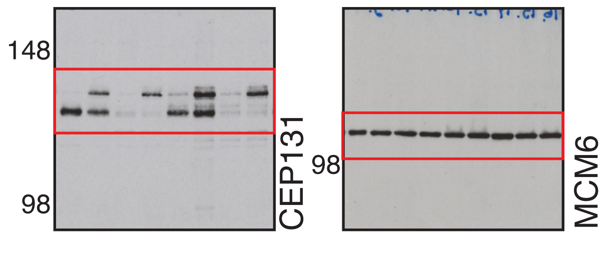

Supplementary Figure 8b

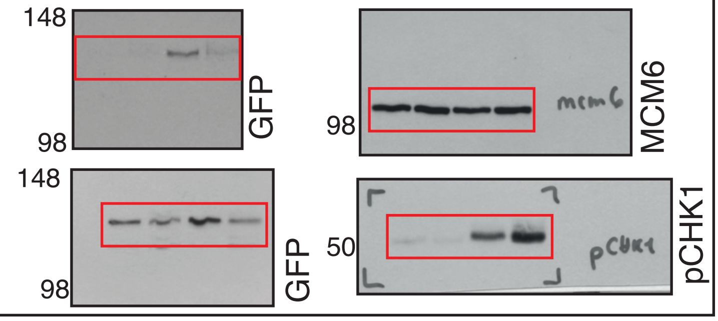

Supplementary Figure 11

Full scans of cropped immunoblots.
